# A genome-scale CRISPR Cas9 dropout screen identifies synthetically lethal targets in SRC-3 inhibited cancer cells

**DOI:** 10.1038/s42003-021-01929-1

**Published:** 2021-03-25

**Authors:** Yosi Gilad, Yossi Eliaz, Yang Yu, Adam M. Dean, San Jung Han, Li Qin, Bert W. O’Malley, David M. Lonard

**Affiliations:** 1 Department of Molecular and Cellular Biology, Baylor College of Medicine, Houston, TX USA; 2 Department of Molecular and Human Genetics, Baylor College of Medicine, Houston, TX USA

**Keywords:** Drug screening, Cancer screening, Breast cancer

## Abstract

Steroid receptor coactivator 3 (SRC-3/NCoA3/AIB1), is a key regulator of gene transcription and it plays a central role in breast cancer (BC) tumorigenesis, making it a potential therapeutic target. Beyond its function as an important regulator of estrogen receptor transcriptional activity, SRC-3 also functions as a coactivator for a wide range of other transcription factors, suggesting SRC-3 inhibition can be beneficial in hormone-independent cancers as well. The recent discovery of a potent SRC-3 small molecule inhibitor, SI-2, enabled the further development of additional related compounds. SI-12 is an improved version of SI-2 that like SI-2 has anti-proliferative activity in various cancer types, including BC. Here, we sought to identify gene targets, that when inhibited in the presence of SI-12, would lead to enhanced BC cell cytotoxicity. We performed a genome-scale CRISPR-Cas9 screen in MCF-7 BC cells under conditions of pharmacological pressure with SI-12. A parallel screen was performed with an ER inhibitor, fulvestrant, to shed light on both common and distinct activities between SRC-3 and ERα inhibition. Bearing in mind the key role of SRC-3 in tumorigenesis of other types of cancer, we extended our study by validating potential hits identified from the MCF-7 screen in other cancer cell lines.

## Abstract (web summary)

Using a CRISPR-based large-scale screen, Gilad et al. identify the genes that show synthetic lethality with an SRC-3 inhibitor in breast cancer cells while contrasting this with the estrogen receptor degrader fulvestrant. This study provides insights into the development of therapeutic strategies for cancer forms that involve SRC-3.

## Introduction

More than 70% of breast cancers (BCs) express the nuclear receptor (NR) estrogen receptor-α (ERα) and are highly dependent on its signaling for tumor growth^1,2^. Therefore, endocrine therapy with either selective estrogen receptor modulators/degraders or aromatase inhibitors is a cornerstone modality in BC treatment. Nonetheless, initial non-responsiveness, as well as acquired resistance in patients with advanced disease, is still an obstacle^2,3^, which makes the search for new therapeutic interventions to treat endocrine therapy-resistance disease highly desired.

Steroid receptor coactivators (SRCs) are critical regulators of NR-mediated gene expression^4^. SRCs are broadly expressed and play key roles in human reproduction and physiology^5,6^ and they are especially important in tumorigenesis^7,8^. Therefore, the importance of SRCs as therapeutic targets cannot be over-estimated, particularly as an opportunity for moving beyond the existing tool-box of BC endocrine therapy, chiefly in cases of acquired resistance which is frequently associated with advanced stages of the disease and gain-of-function mutations in ERα. SRC-3, a member of the SRC protein family, is frequently upregulated in BCs^9–13^ and is associated with poor outcome^14,15^. Recent efforts have been made to meet the challenge of developing small molecule inhibitors for SRC-3 which has been considered a challenging drug target, due to the lack of a high-affinity ligand-binding pocket and the fact that protein–protein interactions largely define its biological activity^5,16,17^. These efforts eventually resulted in the discovery of SI-2, a first-in-class anticancer drug that promotes degradation of SRC-3 and is selectively toxic to cancer cells^18^. Already, it has been shown that SI-2, can be effectively combined with the selective estrogen receptor degrader (SERD) AZD9496 to inhibit tumor growth in an ERα Y537S mutant patient-derived xenograft (PDX) animal model^19^.

SI-12 is a small molecule inhibitor of SRC-3 closely related to SI-2, and to further enhance the efficacy of SI-12 as an anti-cancer therapeutic, we sought to explore a discovery-based approach to identify gene targets that would have synthetic lethality under the pressure of SRC-3 inhibition. A motivation for finding efficient cancer-cell killing partners for SI-12 is to expand its therapeutic window that is frequently small for most cancer drugs and to overcome acquired drug-resistance^20–22^. Establishment of a CRISPR (clustered regularly interspaced short palindromic repeats)-Cas (CRISPR-associated) nuclease system as a feasible high throughput gene-editing technology^23–25^, opened new horizons in drug discovery^26–29^ and dramatically increased the opportunities to explore gene-drug interactions as a platform for identifying synthetically lethal drug combinations^30–33^.

Here we performed a genome-wide CRISPR-Cas9 loss-of-function screen in MCF-7 ER+ BC cells, executed under pharmacological pressure with SI-12, to identify targets whose inhibition will enhance SI-12 anti-tumor activity. Identification of genes whose “dropout” associates with increased sensitivity to SI-12 treatment is the basis for selecting potential candidate targets for combination treatment with SI-12. From this screen, we identified eight candidates for which small molecule inhibitors are commercially available and subsequently validated their cooperative anti-cancer activity in the presence of SI-12 by targeted functional genetics and drug combination experiments. In addition, we found that knockdown (KD) of neuron-derived neurotrophic factor (*NDNF)* and the olfactory receptor (OR) *OR4D6*, screening candidates with no previous reports that link them with tumorigenesis or drug resistance, highly sensitized MCF-7 cells to SRC-3 inhibition. Further exploration on *OR4D6* revealed that additional BC cell lines were also sensitive to its KD in the context of SRC-3 inhibition, pointing to *OR4D6* as a potential anti-cancer therapeutic target and supports the evolving concept that ectopically expressed ORs are hijacked by cancer cells to drive growth factor signaling pathways^34^. By extending the drug-gene vulnerabilities evaluation beyond the MCF-7 cell line, we discovered that highly potent cancer-killing combinations of SI-12 can also be achieved in triple-negative breast cancer (TNBC), pancreatic, and prostate cancer cells with DNMT and RhoA inhibitors.

To shed light on the similarities and differences between the genetic dependencies under ERα versus SRC-3 pharmacological inhibition, we performed an additional screen applying a similar experimental approach, but with the selective ERα degrader (SERD) fulvestrant (ICI) alongside SI-12. Comparison between these screens revealed that along with an expected overlap of some “dropouts”, the two compounds substantially differ in their genetic signatures, which underscores that SRC-3, despite being a key component of the ERα signaling pathway^35–39^, has a variety of other crucial biological roles in cancer cells.

Collectively, by performing these CRISPR-Cas9 dropout screens, we identified a number of potent anti-cancer combinations of the SRC-3 inhibitor SI-12 with small molecule inhibitors for other genes. We utilized seven different cancer cell lines representing four types of cancer to validate the results of our screen through both targeted functional genetics and pharmacological inhibition. Our findings validate SRC-3 as a distinct therapeutic target from endocrine-based therapies and suggest further exploration of ORs as potential targets for intervention in cancer therapy.

## Results

### CRISPR-Cas9 genome-wide screens in MCF-7 cells identifies potential targets for combination anti-cancer activity with SI-12

**Fig. 1:**
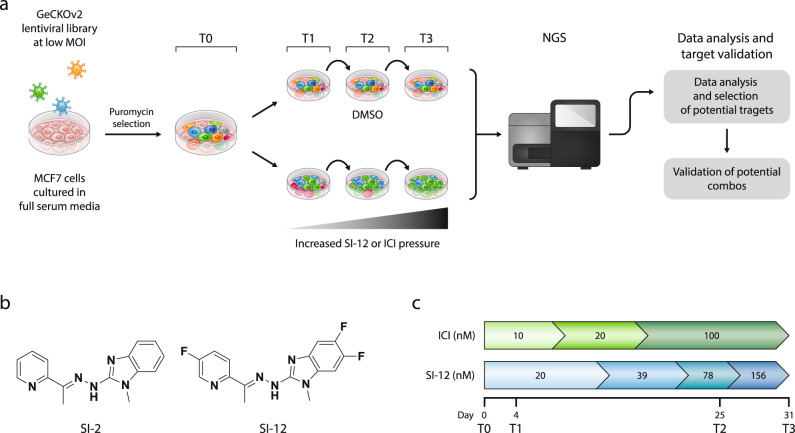
Pooled CRISPR/Cas9 screening using the genome-wide single vector library GeCKOv2 in MCF-7 cells. **a** Schematic outline of pooled CRISPR/Cas9 screens under increased pharmacological pressure of either SI-12 or ICI to identify genetic vulnerabilities for drug combinations. **b** Molecular structures of SRC-3 inhibitors SI-2 and SI-12. **c** Timeline of the screens. Increased drug-pressure was applied on the cells and gDNA was collected at three-time points, which was subsequently amplified by PCR at the bar-coded regions and then subjected to NGS.

To identify genes whose loss of function would substantially increase the sensitivity of ER+ BC cells to SRC-3 inhibition, we performed a genome-wide screen in MCF-7 cells using the GeCKOv2 one vector system library comprised of >120,000 unique sgRNAs (SGR) that target 19,050 genes (Fig. 1a). The plasmid library was acquired from Addgene originally provided from Feng Zheng’s laboratory, amplified, and then packed into a lentiviral vector, following a previously described protocol^40^. MCF-7 cells were infected with the viral library at a low multiplicity of infection (MOI) to minimize the number of cells with more than one genetic editing event. The number of cells at the starting point of the screen was calculated to enable coverage of at least 500 reads per SGR. Twenty-four hours after a viral infection, the cultures were washed and incubated overnight to allow for genetic editing to take place. Untransfected were eliminated by puromycin selection (2 µg/mL, 72 h), after which the resistant cells were washed with PBS, trypsinized, and pooled. An aliquot of the pooled cells was kept for baseline determination (T0), while the rest of the cells were split into three arms; 1—vehicle, 2—SI-12, and 3—ICI (Fig. 1a). SI-12 (Fig. 1b) and ICI cultures were subjected to gradually increasing drug pressure during a period of 31 days (Fig. 1c) to enable selection of resistant populations while minimizing instances of random loss of edited cells as a result of pharmacological stress associated with early use of high drug concentration. Genomic DNA (gDNA) was harvested from the collected cells at three-time points (Fig. 1c), the barcoded sequences were amplified by polymerase chain reaction (PCR) and subsequently purified and sequenced by next-generation sequencing (NGS). To assess the reliability of the NGS reads, Pearson’s correlation coefficient (PCC) was calculated for the baseline to replicate pair (T0, PCC = 0.94) indicating the reliability of the NGS system as well as replicate to replicate reproducibility (Fig. S1a). For assessment of the editing efficiency, we calculated the cumulative distribution fractions (CDF) of SGR abundances for SGRs that target ribosome-related genes and those that target non-ribosomal genes, which shows that at T0 there is only a marginal difference between the two groups. However, at later time points the gap between the CDFs of the two groups was increased, which indicated effective CRISPR-Cas9 gene editing (Fig. S1b).

**Fig. 2:**
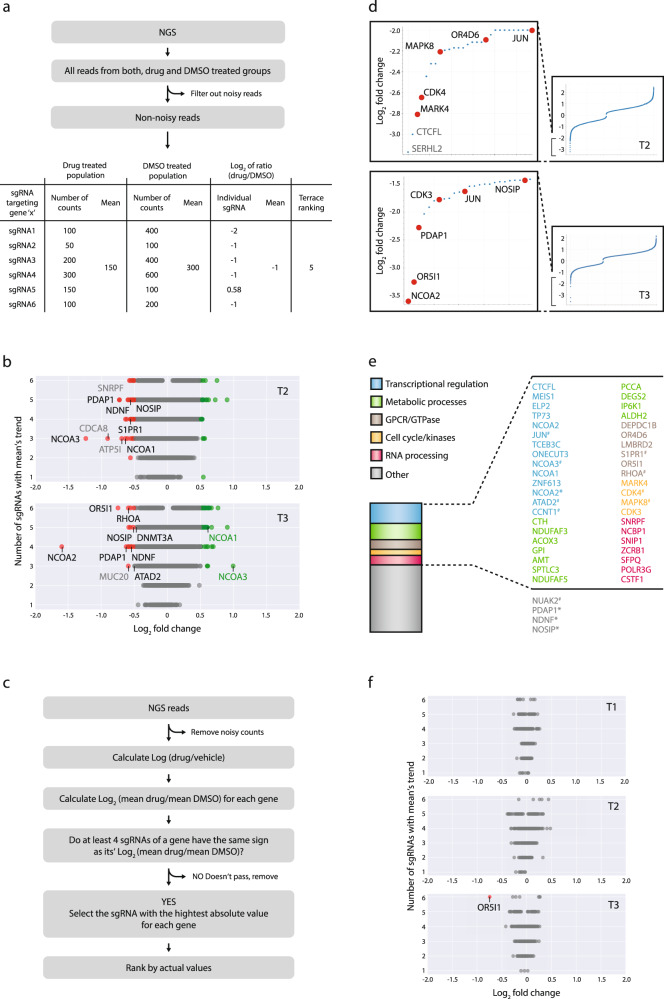
Two strategies, terrace ranking and decisive ranking of CRISPR-screen outputs (DRACO) filtration were applied for the selection of candidate genes. **a** Terrace ranking selection outline (count numbers are arbitrary and were used only for examplification purposes). **b** Top dropped-out genes selected by terrace ranking on terrace plots of time points T2 and T3. **c** DRACO selection outline. **d** Top dropped-out genes selected after applying DRACO algorithm-based filtration. **e** Top ~100 selected candidate genes from T2 and T3 were categorizes based on UniProt primary function indicating that ~50% of the selected genes belong to five major biological processes. #Availability of small molecule inhibitor; *appeared in more than one of the four groups (see (**b**) and (**d**)). **f** Terrace plots for OR genes indicate that OR5I1 is the only OR that is outside the “neutral” distribution.

The on-target specificity and efficacy of the CRISPR-Cas9 gene-editing system, compared to other gene-perturbation techniques, is relatively high^24,41^. Yet, the performance of individual SGRs that are designed to target the same gene can substantially differ^42,43^, which we also observed in our screen (Fig. S1c). Therefore, averaging the effect of a set of SGRs that have the same target gene is usually the method of choice for calculating a KO effect in pooled CRISPR-Cas9 screens. However, considering the fact that in some instances, the efficiency of gene-editing might strongly differ from one SGR to another, in addition to the traditional “average effect” calculation, we applied a terrace ranking method (Fig. 2a, b) as follows: after filtering out all the noisy reads, we calculated the “drug/control” ratio for all the individual SGRs in the library using the following equation: (normalized number of SGR reads in drug-treated population)/(normalized number of SGR reads in vehicle population). After determining the “drug/control” ratios for all the individual SGRs, we calculated their logarithmic values: log_2_ (“drug/control”) as well as the mean logarithmic values for every set-of-six SGRs that have the same target gene: log_2_ (“mean drug/mean control”). Finally, we plotted all the log_2_ (“mean drug/mean control”) values on a terrace chart, ranking from 1 to 6 which reflects the number of individual log_2_ (“drug/control”) values for a given gene that has the same logarithmic sign (+ or −) as the corresponding log_2_ (“mean drug/mean control”) (Fig. 2b).

In order to increase the predictive power of our data analysis, we designed a multi-ranking analytical method that implements previously reported analytical criteria and methodology. We named this ranking method DRACO (decisive ranking of CRISPR outputs), which similar to the “second best” selection strategy^44^, is designed to prioritize potential targets based on a single “drug/control” ratio value (Fig. 2c), rather than relying on the conventional “mean value”-based ranking. The rationale behind using DRACO is to compensate for the lack of uniformity in the editing efficiency of individual SGRs that have the same target gene, which might result in falsely represented phenotypes when relying on a calculated average effect^23,43^. On the other hand, as a method that ranks relying on only one SGR, DRACO inherently lacks a statistical power and to balance this we defined a ‘four out of six’ criteria according to which a potential candidate gene is only considered if at least four out of six of its individual log_2_ (drug/control) values have the same sign (+ or −) as the corresponding mean log_2_ (drug/control) value. After filtering out all the noisy reads as well as genes that did not meet the “four out of six” criteria, the most effective individual SGR per gene was selected as follows: SGR that had the highest absolute log_2_(drug/control) value within its sets was plotted according to its actual log_2_(drug/control) value (Fig. 2d). We believe that target selection applying both methods—terrace ranking and DRACO—reduces the probability of over- or under-estimation of any potential target.

Spread distribution of values at T2 and T3 as compared to T1 (Fig. S2), indicates that the genetic dependencies of the cells increase proportionally to the intensity and duration of the pharmacological pressure. Since the genetic signatures are more apparent at later stages of the screen, we picked the potential candidates from time points T2 and T3 by applying both of the analyses described above. Choosing candidates from two time-separated points, rather than one, might shed light on developing the genetic dependencies and increase the number of potential targets whose inhibition is likely to sensitize resistant cancer cells to treatment with SI-12. By each method, we selected the 25 most dropped-out candidates from T2 and T3, which resulted in a list of ~100 genes whose KO brought about increased vulnerability of MCF-7 cells to SI-12 treatment (Supplementary Data 1, Supplementary 1 Tables). We categorized the selected genes by their main biological function, based on the data available from the UniProt gene ontology database. This analysis revealed that ~50% of the top dropped-out genes belong to five major biological processes; transcription, metabolism, cell cycle, GPCR signaling, and RNA processing (Fig. 2e), which might be attributed to the centrality of SRC-3 in various biological processes including the essential ones listed in Fig. 2e^4,45^. Of note, NCOA1, NCOA2, and NCOA3, which comprise the three SRC family members^46^, were all found amongst the top dropped out genes in the SI-12 screen (Fig. 2b, d, e). This underscores the overlapping roles of these homologous genes^47^ and suggests that their combined biological functions are crucial for cancer cell survival. Intriguingly, in the enriched gene population in the SI-12, T3 group NCOA3 and NCOA1 ranked at #1 and #20 positions (Supplementary Data 1, Supplementary 2 Tables), while at the same time NCOA2 is the most depleted gene in this group (Fig. 2b). This suggests, that except for the known mutually compensatory roles that nuclear co-activators have, adoption of an alternative survival mechanism by a cancer cell during pharmacological pressure might not only make the drug target redundant but in cases of a pleiotropic target such as SRC-3, its loss might be favorable for cancer cell proliferation under a distinct and newly adapted biological state. This assumption is supported by a recently published comprehensive study on endocrine therapy resistance in advanced BC, where for example, MAPK and ER pathways have been shown to fulfill mutually exclusive tumor-growth associated functions in separate metastatic lesions from a single patient^48^. Our unique observation points out that a possible replacement of one pro-oncogenic program by another, as a result of drug-selection pressure, not only makes the drug target redundant but that its loss might produce favorable conditions for cancer cell proliferation under a distinct and newly adapted biological state.

From the top 100 dropped-out genes, eight targets for which small-molecule inhibitors are available were selected for further evaluation (Table S1). There is no molecule available for direct targeting of CCNT1, however, CCNT1 is the cyclin partner of CDK9 in the P-TEFb complex^49^, hence we included the CDK9 inhibitor—atuveciclib (Atuve) as a potentially effective drug for combination with SI-12. Interestingly, we found that two out of seven genes that comprise the GPCR signaling group are ORs (OR5I1, OR4D6). ORs is the largest category of receptors within the GPCR superfamily and its members are emerging as novel targets for cancer therapy^34,50^. In BC, the OR genes OR2B6 and OR2W3 have been suggested as potential biomarkers for disease progression^51^. In order to assess if there is the a genetic dependency of MCF-7 cells for OR family genes under the conditions of the screen, we plotted only OR genes on a terraced plot by filtering out all rest of the genes (Fig. 2f). Confinement of all ORs, except for OR5I1, to the neutral “gray area”, indicates that there is no broad oncogenic reliance on multiple OR-mediated GPCRs, but that the ectopic expression of a specific OR may suffice to promote cancer cell growth. This suggests that among a wide range of OR genes, a single member may drive GPCR signaling that can promote cancer cell proliferation, which is supported by recently published studies in breast and prostate cancers^52,53^.

### Target validation in MCF-7 cells

**Fig. 3:**
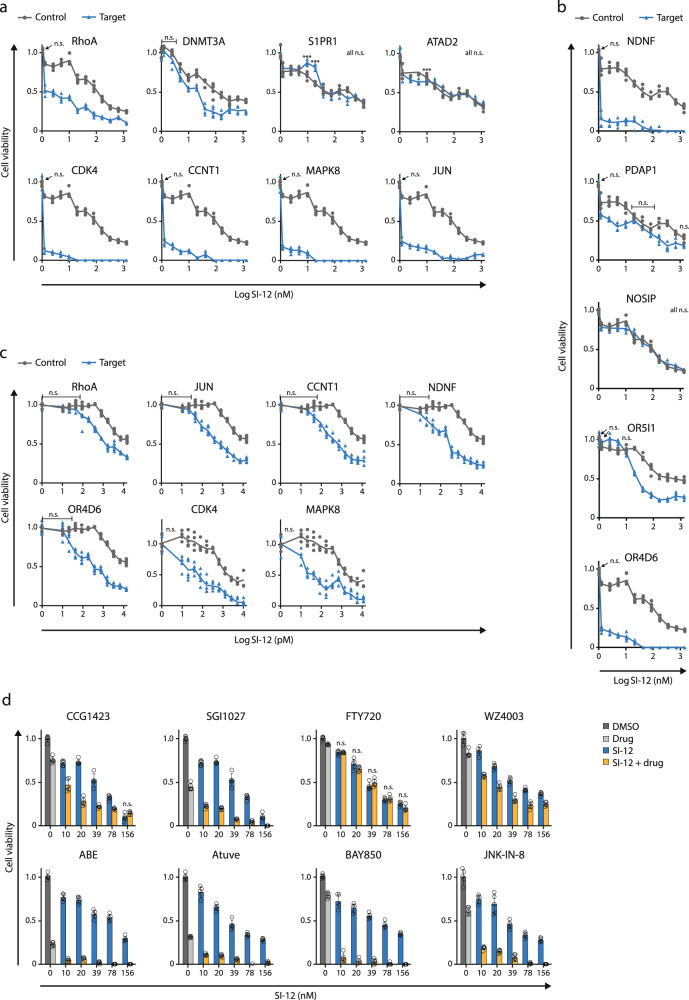
Validation of potential targets by siRNA gene perturbation and drug combination in MCF-7 cells. **a** Set of eight hits for which small-molecule inhibitors are commercially available were evaluated individually by siRNA gene perturbation. **b** Five genes without available small-molecule inhibitors were individually validated by siRNA gene perturbation :NDNF, NOSIP, and PDAP1 are genes that appeared in more than one group of the four 25 gene groups that comprise the top 100 dropout candidates (Supplementary Data 1, Supplementary 1 Tables). OR5I1 and OR4D6 belong to a large multigene family of olfactory receptors that are evolving as potential targets in cancer therapy. **c** For Rhoa, Jun, CCNT1, NDNF, OR4D6, CDK4, MAPK8 genes a second round of siRNA perturbation experiments  was performed with lower concentrations of SI-12. In all the siRNA perturbation experiments the cells were treated with 10 nM of the indicated target siRNA or negative control siRNA (NC) for 48 h, plated in 96 well plates, and exposed to SI-12 treatment for 96 h. At the end of the SI-12 treatment period, the cells were subjected to MTS viability assay. **d** In vitro drug combination experiments. Small molecule inhibitors that target the screen hits were tested in combination with SI-12. Cells were plated in 96 well plates and treated with the indicated compound(s) for 96 h. At the end of the drug treatment period, the cells were subjected to an MTS viability assay. For drug concentrations see Table 1. Each point reflects at least four technical replicates. Each cell viability plot represents at least two independent experiments showing similar results. Statistical significance compares between the combo and the most effective single agent (either SI-12 at relevant concentration, or the partner molecule). ***For all the results *P* < 0.01, two-tailed Student’s *t*-test, if not mentioned otherwise. *n.s*., not significant.

The selected screening candidates were individually validated by siRNA perturbation and pharmacologically—by drug combination cytotoxicity assays. In addition to the targets listed in Table S1, for which commercial inhibitors are available, we included five other candidates from the top 100 dropped-out genes list (Supplementary Data 1, Supplementary 1 Tables) in our siRNA experiments: NDNF, NOSIP, and PDAP1, since they appeared in more than one of the four 25 gene groups (Supplementary Data 1, Supplementary 1 Tables), and two OR genes, OR5I1 and OR4D6, due to recent evidences that potentiate ORs  as targets in cancer therapy^34^. MCF-7 cells were pre-treated with the indicated siRNA and then subjected to SI-12 treatment. KD of 10 out of 13 genes significantly increased the vulnerability of MCF-7 cells to SI-12 treatment (Figs. 3a–c and S3), which indicates the predictive power of the screen and candidate selection strategy. In the cases of RhoA, Jun, CDK4, CCNT1, MAPK8, NDNF, and OR4D6, pre-treatment with siRNA brought about extremely high sensitivity to SI-12 treatment even with the highest titration of the drug (Fig. 3a, b). Therefore, for these genes, an additional set of siRNA perturbation experiments was performed with exposure to lower doses of SI-12 post-siRNA treatment (Fig. 3c).

KD of SRC-1, SRC-2, and SRC-3 also increased the sensitivity of the cells to SI-12 (Fig. S4a), which not only provides additional support to the validity of the screen but also highlights the compensative nature and overlapping biological functionality of the SRCs. As opposed to the majority of the selected targets, that have established oncogenic roles, NDNF and OR4D6, to the best of our knowledge, have no previously known association with cancer progression or drug resistance. It, therefore, was intriguing to us that KD of these genes resulted in a dramatic enhancement in the cytotoxicity of SI-12, suggesting the further exploration of their role in tumorigenesis.

**Fig. 4:**
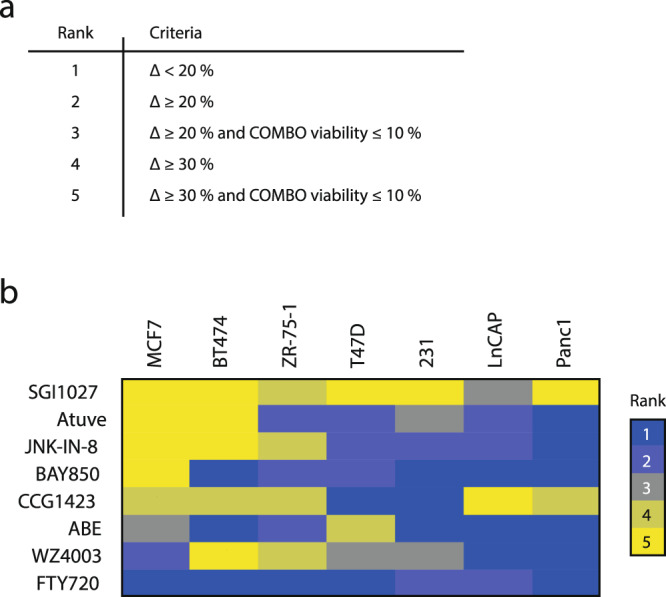
Combo efficacy ranking. **a** Criteria associated with its rank. The ranking range is 1–5, where 1 represents the lowest level of additive killing effect and 5 represents the highest. **b** Eight compounds tested in combination with SI-12 on seven cancer cell lines (Figs. 3d, 5, and 6). The additive killing effect was heat-mapped according to the combo efficacy ranking in (**a**).

An additional iteration for target validation was performed by drug combination (combo) experiments.  First, the bioactive concentrations of the individual compounds in MCF-7 cells were assessed (Fig. S5a), followed by combo experiments.  Combo efficacacy was compared to the efficacy of the equimolar concentrations of single agents (Fig. 3d). To rank the additive killing effect of each drug combination we defined five hierarchical levels of efficacy, where level 1 represents the lowest additive killing effect (it is when the gap between the killing effect of a single drug and the combo is <20%) and level 5 represents the highest additive killing effect (it is when the gap between the killing effect of a single drug and the combo is ≥30%) (Fig. 4a) .Any drug combination was considered as “effective” only if it possessed a significantly higher killing effect compared to the most potent individual drug. Six out of eight tested combinations showed a substantial additive killing effect with an efficacy level of ≥3 (the gap between the killing effect of the most potent n individual drug and the combo is ≥20%) (Fig. 4b), which supports our results from the siRNA perturbation experiments and reinforces the reliability of our candidate selection strategy.

Importantly, no additive killing effect on primary mouse hepatocytes was observed for any of the above-tested drug combinations (Fig. S5i and Table S2), which suggests predominant toxicity to malignant cells rather than normal tissues. However, SGI1027, FTY720, and WZ4003 by themselves showed greater than EC_50_ activity toward primary hepatocytes. As SGI1027 represents one of the most effective combinations with SI-12, we tested an alternative DNMT inhibitor, CM272. CM272 which substantially increased the cancer cell killing effect in combination with SI-12 (Fig. S6), yet it was not toxic to the hepatocytes (Fig. S5j and Table S2), which suggests that DNMT as a atrget should not be ruled out for improved cancer cell killing in combination with SRC-3 inhibition.

Next, by taking advantage of the availability of additional alternative small molecule inhibitors we performed a secondary validation for another three biological targets; CCG203971 (Rhoa inhibitor), DB07268 (JNK inhibitor), and Palbociclib (CDK4/6 inhibitor). In all the cases a significant enhancement in the killing effect of MCF-7 cells, compared to single-agent activities, was observed (Fig. S6), which affirms the validity of these targets as effective drug combinations with SI-12.

As mentioned above, most of the selected candidates have known associations with tumorigenesis^54–63^. For instance, CDK4 and ATAD2, are known to be important in ER+ BC; CDK4 is a clinical target for the treatment of ER+ BC^64,65^ and ATAD2 has been characterized as a marker for poor prognosis in several types of cancers (Fig. S7). Both of these genes are thought to play critical roles in the tumorigenesis of hormone-dependent diseases, including BC^66,67^, due to their roles as ERα and AR coactivators^68,69^. ATAD2 has been directly associated with SRC-3 where it has been identified as both an SRC-3 target gene and SRC-3 associated histone acetyltransferase^67^. In addition, the probable convergence of ATAD2 on SRC-3 might explain why the inhibitor of ATAD2, BAY850, possessed a relatively low additive cancer cell killing effect in combination with SI-12 across seven out of eight tested cell lines with maximal gap of 30% between the killing effects of a single drug and the combo (Fig. 4b).

### Assessment of SI-12 drug combination effectivity in other cell lines

**Fig. 5:**
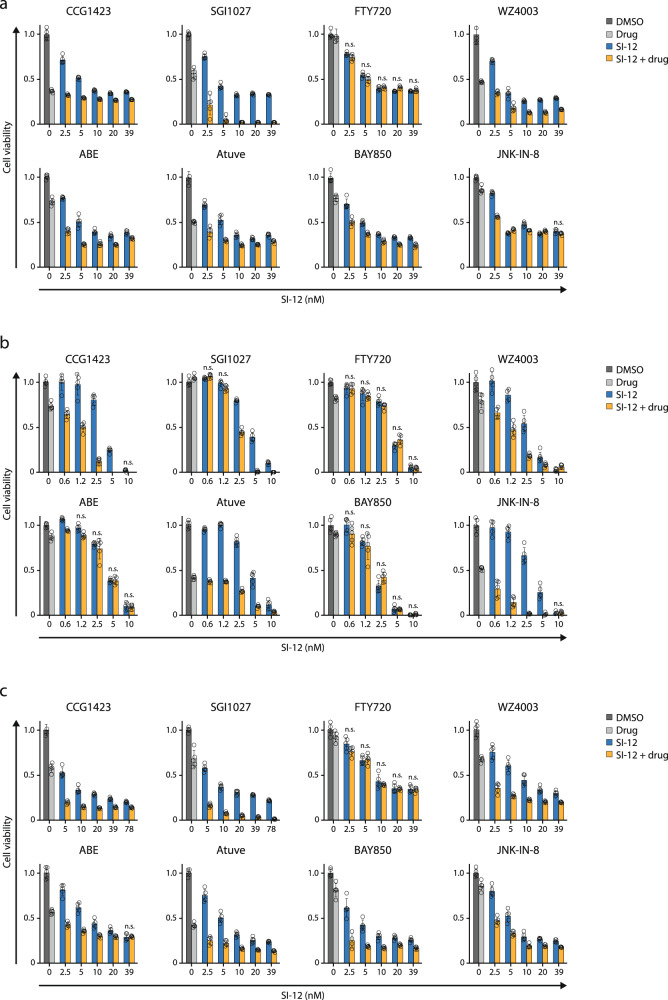
In vitro drug combinations experiments in ER+ BC cell lines. **a** In T47D cells. **b** In BT474 cells. **c** In ZR-75-1 cells. Cells were plated in 96 well plates and treated with the indicated compound(s) for 96 h. At the end of the drug treatment period, the cells were subjected to an MTS viability assay. For drug concentrations see Table 1. Each point reflects at least four technical replicates. Each cell viability plot represents at least 2 independent experiments showing similar results. Statistical significance compares between the combo and the most effective single agent (either SI-12 at relevant concentration, or the partner molecule). ***For all the results *P* < 0.0005, two-tailed Student’s *t-*test, if not mentioned otherwise. *ns*, not significant.

**Fig. 6:**
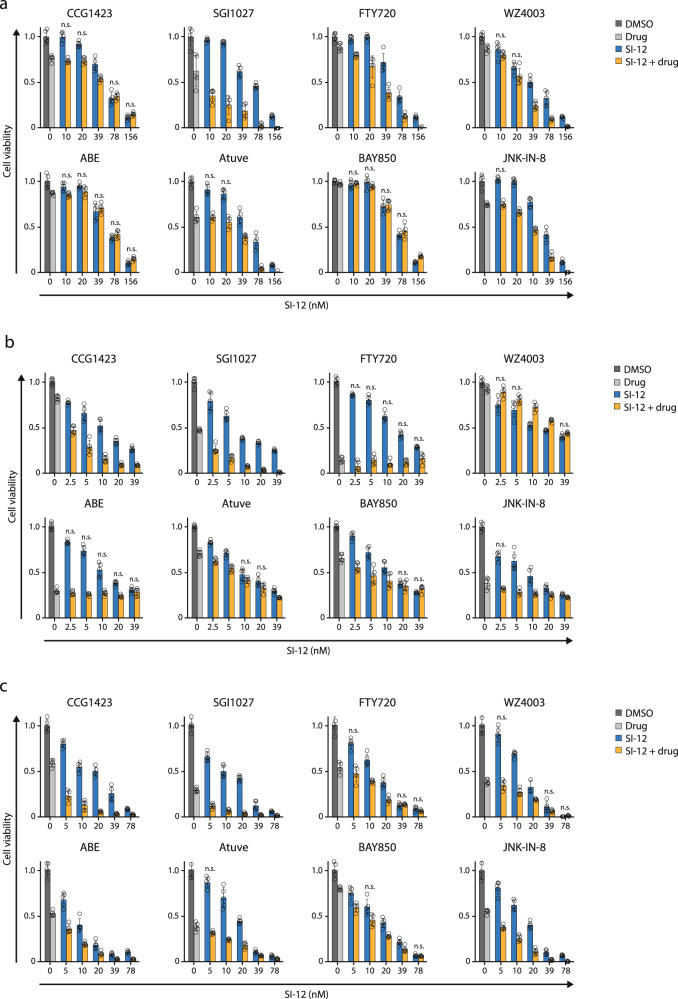
In vitro drug combinations experiments in non-ER+ cancer cell lines. **a** In TNBC cell line MDAMB-231. **b** In pancreatic cancer cell line PANC-1. **c** In prostate cancer cell line LNCaP. Cells were plated in 96 well plates and treated with the indicated compound(s) for 96 h. At the end of the drug treatment period, the cells were subjected to an MTS viability assay. For drug concentrations see Table 1. Each point reflects at least four technical replicates. Each cell viability plot represents at least two independent experiments showing similar results. Statistical significance compares between the combo and the most effective single agent (either SI-12 at relevant concentration, or the partner molecule) ***For all the results *P* < 0.0005, two-tailed Student’s *t*-test, if not mentioned otherwise. *ns* not significant.

Considering the cost and effort that is invested in genome-scale screenings, the ability to infer from studies that are performed in one cancer cell line to cell lines representing other cancer types is highly valued. Therefore, for assessing to what extent the combinations that were tested in MCF-7 cells might reflect on general drug combination efficacy in ER+ BC, as well as for obtaining information regarding the potential pan-cancer sensitivity, we evaluated the screen-selected targets in three additional ER+ BC cell lines; ZR-75-1, BT-474, and T-47D; a TNBC cell line, MDA-MB-231; and two non-BC cell lines, LNCaP (prostate) and PANC1 (pancreas). Identifying a cooperative killing effect by combining lower doses of single agents, rather than using a high dose of either compound, was sought to achieve the greatest cytotoxicity toward cancer cells in a way that should maximize the drugs’ therapeutic window. Therefore, after we assessed the toxicities of single compounds across the tested cell lines (Fig. S5b–g), we performed combo experiments where the concentration of SI-12 was titrated and the concentrations of the “partner” compounds were preferably kept above their IC50 levels (Figs. 5 and 6; Table 1). Among each of the cell lines mentioned above, two ER+ lines - BT-474 and ZR-75-1 - were found as the overall most responsive to the combinational treatments (Fig. 4b). This observation bolsters the predictive power of our screening strategy and candidates selection and suggests that these drug combinations are likely going to be effective for use in ER+ BC . Targeting DNMT, and in the majority of cases also RhoA, in combination with SRC-3 inhibition, was effective across the cancer lines that have been tested. Interestingly, the *NUAK2* inhibitor, WZ4003, contributed to the anti-cancer activity of SI-12 across seven out of eight tested cell lines but possessed a moderate rescue-like effect in PANC-1 cells. This observation was found to correlate with the data from “The Human Protein Atlas” showing that NUAK2 is a favorable prognostic marker in pancreas cancer (Fig. S7b).

**Table 1:** Summary of substances concentrations in µM for Figs. 3d, 5, and 6.

Cells	CGG1423	SGI1027	FTY720	WZ4003	ABE	Atuve	BAY850	JNK-IN-8
MCF7	10	5	2.5	5	2.5	0.625	2.5	2.5
ZR-75-1	10	2.5	5	10	1.25	1.25	5	2.5
BT474	10	0.156	5	10	1.25	0.625	5	2.5
T47D	10	1.25	5	10	5	1.25	5	5
MDAMB231	10	1.25	5	10	0.078	0.156	0.312	2.5
LnCAP	10	0.625	5	10	1.25	0.312	1.25	5
Panc1	10	2.5	5	10	1.25	1.25	5	5

*OR4D6* and *NDNF* are two targets that do not have an available small molecule inhibitor, yet their inhibition with siRNA resulted in high sensitization of MCF-7 cells to SI-12 treatment (Fig. 3b, c), which was intriguing to us since to the best of our knowledge these genes have no previously published association with cancer progression. Therefore, for assessing whether KD of these genes isspecifically effective for sensitizing onlyMCF-7 cells to SI-12 treatment, we performed siRNA perturbation experiments in four additional BC cell lines (three ER+: BT474, ZR-75-1 and T47D, and one TNBC: MDAMB-231). When compared with MCF-7 cells, the contribution of *OR4D6*  or *NDNF* KD to SI-12 sensitization in these cells lines was relatively moderate. Nonetheless, three of the tested cell lines become more sensetive to SI-12 treatment  as a result of *OR4D6*KD and one was sensitive to KD of *NDNF* (Fig. S4b). These results suggest  that *NDNF* might be a cell line-specific sensetizor to SRC-3 inhibition, while OR4D6 should be considered as a potential therapeutic target in BC in general.

Overall, by extending our evaluation to cell lines representing a range of cancer types, we were able to identify pharmacological combinations that can potentially be applied across different cancers.

Of note, across all the tested cell lines, the sphingosine-1-phosphate receptor (S1PR) modulator Fingolimod (FTY720), when used at high concentrations, showed a strong single-agent anti-proliferative potential (Fig. S5h). This observation is in agreement with previous publications, where the anti-neoplastic side effects of FTY720, via processes other than S1PR signaling^70^, were investigated^70–74^, supporting the potential value of repurposing this FDA-approved immunosuppressant drug (for MS)^75^ as an anti-cancer agent.

### Combinational treatment with SI-12 and other small-molecule drugs improved the growth inhibition of BC organoids

**Fig. 7:**
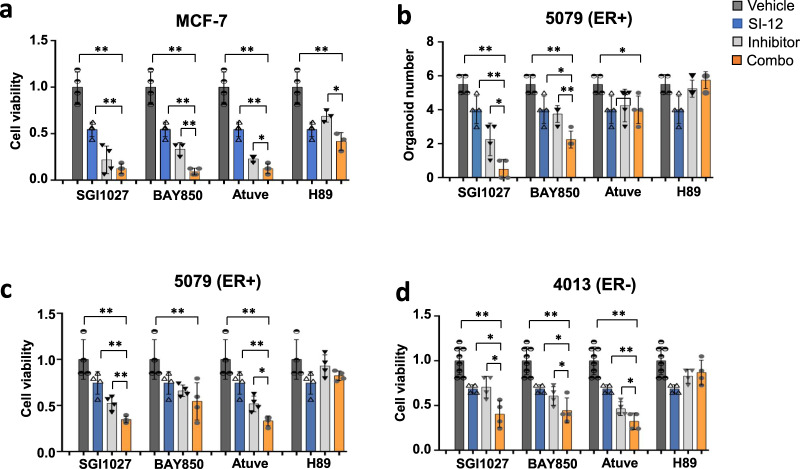
Drug combination experiments in cancer organoid models. **a** Cell viability in MCF-7 cells-derived organoids. **b** The number of PDX 5079 organoids in each well was counted after 2 weeks of treatment. **c** Cell viability in ER+ PDX 5079 organoids. **d** Cell viability in TNBC PDX 4013 organoids. All the experiments were performed for 2 weeks after which the organoid and cell number were counted. Fresh medium with or without drugs was provided every 3–4 days. ** *P* < 0.01, *P < 0.05 two-tailed Student’s *t*-test.

Organoid culture is an in-gel model system that uses normal or tumor epithelial cells that can recapture the complex composition of tumors and has become an emerging tool for drug screening and testing^76,77^. We implemented this model system  for further evaluation of SI-12 combos with either SGI-1027, BAY850 or Atuveciclib, which represent three of the most potent candidates from our screen (Fig. 3d), while H89 was used as a negative control since its combination with SI-12 was mostly not effective (Fig S5k and Table S3). Four different BC organoid cultures were treated with 50 nM SI-12 alone or in combination with either SGI-1027, BAY850, Atuve, or H89 for 2 weeks (Fig. 7). BAY850 and Atuve combinations with SI-12 in MCF-7 organoids resulted in a significant additive cancer cell killing effect (Fig. 7a), which directly reflects the results that were obtained from monolayer cell culture experiments (Fig. 3d). The strongest inhibition of organoid formation and growth was observed with combined SI-12 and SGI-1027 treatment in the 5079 ER+ organoid line (Fig. 7b, c). Compared with vehicle treatment, this combination treatment led to a more than 90% decrease in organoid number which is substantially more effective than SI-12 alone (30% decrease) or SGI-1027 alone (40% decrease) treatments (Fig. 7b). Consistent with these results, cell viability in the 5079 line was reduced by more than 60% with  a combo, but only declined by 25% and 50%, respectively in the SI-12 and SGI-1027 single  drug treatments (Fig. 7c). Similar results were observed in the triple-negative 4013 organoid line: a combo of SI-12 and SGI-1027 strongly reduced the cell viability compared to single drug treatment with either SI-12 or SGI-1027 (Fig. 7d). These results indicate that combined SI-12 and SGI-1027 treatment can achieve stronger inhibition on TNBC organoid formation and growth than single treatment with either compound, which solidifies our observations from cell culture experiments that suggested a pan-cancer effective nature of this combination (Figs. 3d and 5a–c).

### SRC-3 and ERα targeting agents have overlapping but distinct genetic vulnerability signatures

**Fig. 8:**
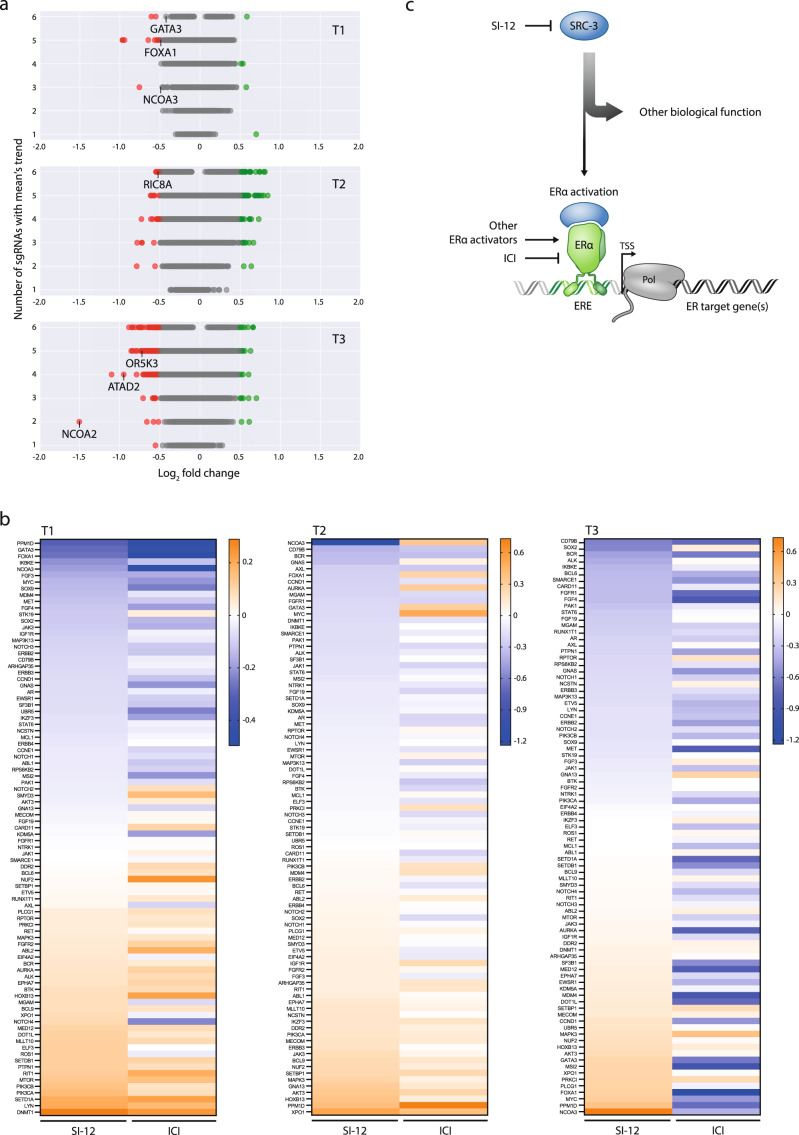
Distribution of top BC oncogenes in ICI and SI-12 screens is compared. **a** Terrace plots of ICI screen. **b** Comparison of top oncogenes from ICI and SI-12 screens. A list of top BC oncogenes was ranked according to their dropout/enrichment status in the SI-12 group for each of the time points T1, T2, and T3. Following that, a column that is comprised of the same list of genes in the ICI group was adjacently aligned. Lastly, the data was visualized using a heat-map representation. The list of the top BC oncogenes is comprised of top mutated and amplified genes in BC, based on data from TCGA. The total number of samples is 976 and 1093, respectively. Calculations of Pearson/Kendall/Spearman correlations between the distributions of the genes in both screens: T1—0.90/0.70/0.86, T2— 0.50/0.49/0.66, T3—0.51/0.35/0.49, respectively. **c** Diagram showing that ERα and SRC-3 are overlapping, but not identical therapeutic targets.

SRC-3 is a key regulator of the transcriptional activity of ERα^38,39^. In order to assess the degree of overlap and distinction between gene vulnerability signatures under conditions of pharmacological inhibition of either ERα or SRC-3, we performed a parallel CRISPR-Cas9 drop-out screen with ICI (as outlined in Fig. 1a, c). We applied the same strategy for the selection of the top 100 dropped out genes under ICI pressure as we did for SI-12 (Figs. 8a and S8a, b). Importantly, three prominent ER signaling pathway genes, FOXA1 GATA3 and NCOA3, were rapidly depleted (T1, Fig. 8a). Of note, this finding is in agreement with the results from a recently published study, where an alternative CRISPR library was used to perform a similar screening using MCF-7 cells cultured in full serum media^78^ substantiating our screen’s validity. Comparison between the top 100 dropped-out genes from the ICI screen to the top 100 dropped-out genes from the SI-12 screen reveals ~12% of overlap (12 genes) (Fig. S8c), including ATAD2 that is known as a key regulator of ERα transcriptional activity^79^ and CDK4, which even though is not directly associated with ERα signaling, is known as a therapeutic target for ERα inhibitor-resistant BC^80–82^. To obtain additional insight into the comparison between ERα and SRC-3 inhibition-related genetic dependencies, we mined the cancer genome atlas (TCGA) genomic datasets to compare between the appearances of top BC oncogenes in the SI-12 and ICI groups. For this purpose, we created a list of top BC oncogenes, based on TCGA data of the top ~100 mutated/amplified genes in BC (Supplementary Data 1, Supplementary 3 Tables) after which, for each of the three-time points (T1, T2, and T3) we listed these genes in a descending order according to their dropout/enrichment level in the SI-12 group. Then, we aligned an adjacent column that is comprised of the same list of genes and thier associated values  in the ICI group. Finaly the two columns were visualized in the form of a heat map (Fig. 8b, Supplementary Data 1, Supplementary 4 Tables). This analysis reveals the differential interaction of the two drugs with BC signature oncogenes. Moreover, the Pearson correlation between the distribution of these genes in the SI-12 and ICI groups at T1 is higher compared to the later time points T2 and T3, 0.78, 0.27, and −0.03, respectively (Fig. 8b). This observation is not surprising, since, at the early stages of the screen, the specific effects of either drug are not expected to be entirely emergent. However, after prolonged exposure and continuously increasing concentrations of SI-12 and ICI, the impacts of the drugs and the differences between their interactions with specific genes become apparent (Fig. 8b). The differences in SRC-3 as a molecularly distinct target from ERα, manifest as well in the relatively low dependence of the SI-12 treated group on key ER pathway genes as compared to the ICI treated group, e.g., MYC at time point T1, FOXA1 at T2, MYC, and GATA3 at T3 (Fig. 8b). In a recent publication by Xiao et al., a whole-genome CRISPR-Cas9 screen was conducted in ER+ BC cells to identify key regulators of endocrine resistance^83^. The authors found strong depletion of ER pathway-related genes, such as ESR1, GATA3, FOXA1, MYC, and NCOA3, in the estradiol treated group. These findings are well aligned with what is known about the ER signaling network and are expected within the experimental conditions that included the use of stripped serum culture media. In our study, a relatively modest dropout of ER pathway genes was associated with anti-estrogen treatment. The primary reason for this difference between the two screens is probably attributed to the different experimental conditions, as our screen was performed in a full serum media in order to better mimic physiological conditions that include diverse estrogen-independent growth factor pathways acting on tumor cells.

Despite an expected similarity between SI-12 and ICI, a distinction in the pharmacological signatures for each compound highlights the fact that SRC-3 has various biological functions other than its role as an ERα coactivator, which underscores its distinctiveness as a pharmacological target.

## Discussion

Technological advancements in CRISPR-Cas9 based gene targeting has enabled genome-wide loss-of-function screening as a powerful platform to explore drug resistance in cancer and to discover effective anti-cancer drug combinations^27,30,52,84–88^. In this study, we utilized the GeCKOv2 CRISPR-Cas9 library to identify novel molecular targets for combination therapy with the SRC-3 inhibitor SI-12 in ER+ BC. For the identification of genes that are most likely to support cancer cell survival during pharmacological inhibition of SRC-3, we subjected MCF-7 cells to prolonged treatment with SI-12 while gradually increasing its concentration (Fig. 1c).

To achieve maximal accuracy in hit selection, we used two different methods to rank the candidates, while for minimizing the number of false discoveries, each method identified not only the magnitude of the measured phenotype, namely enrichment or depletion as a result of gene KO but also its statistical significance. This was achieved by setting a criterion, according to which a certain gene could be considered as a candidate only if at least four individual SGRs from set-of-six SGRs that target the gene, are required to produce the same phenotype (i.e., enrichment or depletion) as the average value of all the set. At the same time, in order to minimize the number of relevant hits that might be neglected due to the “neutralizing” nature associated with the “average value”-based ranking, we designed DRACO—an analytical method that ranks according to individual highly dropped-out SGRs rather than by average values (Fig. 2a, c).

The dominant presence of NCOAs 1–3, among the top-ranked dropout SGRs after prolonged exposure to SI-12 (T2 and T3), provides strong support for the validity of our candidate selection methodology and likely reflects the essentiality of residual and compensatory activities between all the NCOAs when drug pressure is applied. On the other hand, the presence of NCOA3 and NCOA1 amongst the most enriched genes at T3, suggests that persistent pharmacological pressure might result in target redundancy. We speculate that in cases of a pleiotropic target such as SRC-3, drug target loss and the shift to an alternative tumor escape pathway might even be favorable for cancer cell survival.

In the majority of cases, individual inhibition of the selected candidates by genetic and pharmacological methods resulted in an increased cancer cell killing by SI-12, proving the effectiveness and accuracy of our strategy (Fig. 3). Furthermore, drug combination experiments that were performed in three additional ER+ BC cell lines, other than MCF-7, showed that five out of eight combinations were effective in at least one additional ER+ BC cell line (Figs. 4b and 5). These results provide additional support to the validity of the screen-based selected drug combinations and suggest their further clinical development for ER+ BC tretment.

Since the role of SRC-3 in tumorigenesis is not restricted to its main biological function as ERα coactivator^89,90^, we decided to test the therapeutic benefit of drug combinations that have been discovered in the MCF-7 cells in three additional cancer models. Though most combinations that we have tested might be predicted to be relevant only for ER+ BC cells, several combinations were found to be effective in TNBC (MDAMB-231), pancreatic (PANC-1), and prostate (LNCaP) cancer models as well (Fig. 6). For instance, we found that the targeting of DNMT in combination with SI-12 was effective across all the cancer types that we have tested, which highlights the potential of DNMTs as therapeutic targets in combination with SRC-3 inhibition, likely expanding the clinical application for DNMT inhibitors beyond hematologic malignancies^91,92^. Similar to the pan-cancer activity demonstrated by a combination of SI-12 with the DNMT inhibitor SGI1027, combining SI-12 with the RhoA inhibitor CCG1423 also resulted in potent anti-cancer activity in PANC-1 and LNCaP cells in addition to that seen in MCF-7 cells. The anti-cancer activity of this combination, along with the fact that GPCR-signaling related genes comprised seven out of the top one hundred drop-out candidates, underscores the importance of GPCR signaling components as targets in cancer drug-combination therapy^93–95^. Particularly, our findings point to GTPase signaling targets as highly relevant for achieving synthetic vulnerability in SRC-inhibited cancer cells^96^, as supported by previously published studies^97–99^. Interestingly, we found that RhoA inhibition was predominantly effective in LNCaP prostate cancer cells, which aligns with the previously demonstrated importance of RhoA in this type of malignancy and suggests combined co-inhibition of RhoA and SRCs as a potential treatment for prostate cancer^100,101^.

In addition, SI-12 combination treatments with either BAY850 or Atuve also showed improved inhibition on the growth of both the 5079 and 4013 organoid lines, compared to single treatment (Fig. 7b–d). Combined SI-12 and H89 treatment did not show substantial improvement in either the 5079 or 4013 organoid lines compared to single treatment with either compound, indicating that H89 is not a candidate for effective cancer cell killing combination with SI-12, as also suggested by cell culture experiments. On the other hand, a combination of SI-12 with the DNMT inhibitor SGI1027 showed significant inhibition in two out of the three tested organoids, 5079 and 4013, which represent ER+ and TNBC types respectively, reflecting the pan-cancer effectiveness of this combination, as observed in cell culture experiments. Importantly, SI-12 combined treatment with either BAY850 or Atuve, the two most potent combinations found in the MCF-7 cells, showed a significant additive killing effect in the MCF-7 organoid model as well (Fig. 7a). In summary, we found that several combination treatments with SI-12 successfully inhibited the growth of organoids, like that seen in cell culture models, which provides additional support to the validity of our hit selection strategy.

As an inhibitor of a key coactivator for ERα^36–39^, SI-12 could be considered as an analog to anti-estrogen-based endocrine therapy. However, this possibility should be weighed against the fact that SRCs can drive the activity of a wide range of transcription factors in addition to ERα and other nuclear receptors^89,90^. In order to compare the pharmacological signature of SI-12 to that of ERα inhibition, we performed a parallel CRISPR-Cas9 screen using ICI in place of SI-12 (Fig. 1a, c). Comparing the impact of the two substances on top BC oncogenes revealed that aside from some overlap, there is a substantial distinction between their genetic signatures (Fig. 8b). These results affirm, that in addition to the well-established role of SRC-3 in the ERα signaling pathway, SRCs are distinct therapeutic targets. Indeed, we have already shown that when used in combination with a selective estrogen receptor degrader, the closely related SRC-3 small molecule inhibitor, SI-2, can block tumor growth in an ESR1 mutant PDX model system^19^. These findings thus establish SRC-3 as a distinct pharmacological target that is expected to complement but not duplicate existing endocrine therapies (Fig. 8c).

Overall, we discovered potential drug combinations with SI-12 by performing a genome-wide CRISPR-Cas9 screen in MCF-7 cells. Most of these combinations were effective in other ER+ BC cells as well. Substantial additive killing effect with SI-12 was achieved by siRNA inhibition of *OR4D6* and *NDNF*, two genes that had no previous association with tumorigenesis. This finding exemplifies the potential of CRISPR-Cas9 genome-wide screens to discover potential targets for therapeutic intervention and underscores the evolving role of OR genes as targets for cancer therapy. Furthermore, our screen in MCF-7 cells was useful for the discovery of pan-cancer potent combinations, as demonstrated by combinations of SI-12 with either DNMT or RhoA in PANC-1, LNCaP, and MDAMB-231 cancer cell models. Finally, by performing a parallel screen, in which we used the ERα degrader ICI in place of SI-12, we showed that these two molecules impose distinct genetic selection signatures on the cells. This comparative study establishes SRC-3 as a distinct target among existing endocrine therapies.

## Methods

### Cell lines and reagents

All cell lines but 293FT were obtained from ATCC and regularly checked for mycoplasma. MCF-7, BT-474, MDA-MB-231 and PANC-1 cells were maintained in full DMEM medium (10% fetal bovine serum [FBS], 1% Glutamax (ThermoFisher 35050061) and 1% penicillin/streptomycin).

T-47D, ZR-75-1 and LNCaP cells were maintained in full RPMI medium (10% FBS, 1% Glutamax (ThermoFisher 35050061) and 1% penicillin/streptomycin). 293FT cells were obtained from ThermoFisher (R70007) and maintained in full DMEM media at low passage (10>). All cultures were maintained at 37 °C under a 5% CO_2_ atmosphere.

SI-12 was synthesized according to the previously described procedure^18^. In brief: SI-12 4-fluoroacetophenoe was reacted with 5,6-difluoro-2-hydrazineyl-1H-benzo[d]imidazole^18^. The final product was purified and characterized by NMR and HPLC-MS. SGI1027, CCG1423, Fingolimod, Abemaciclib, Atuveciclib, BAY850, JNK-IN-8, WZ4003, were purchased from MedChemExpress.

### Cancer cell viability assay

Cells were seeded at high cell density in 96 well plates and allowed to adhere overnight (MCF-7, LNCaP, PANC-1-10K cells/well; T-47D, BT-474-20K cells/well; MDA-MB-231-3K cells/well). Following media removal, the cells were provided with fresh drug or vehicle-containing media. After an indicated period of drug/compound treatment, the drug/compound-containing media was replaced by fresh media supllied with the MTS reagent (Promega—CellTiter 96 AQueous One Solution). Following this, the cells were returned to the incubator for an additional 1–4 h. Absorbance values were obtained using a Multiskan FC Microplate Photometer plate reader (ThermoFisher) at 490 nm. After subtraction of the media-only read (blank) from all the actual MTS reads, cell viability was calculated relative to vehicle-treated cells. Each point reflects at least four technical replicates. Each cell viability plot represents at least two independent experiments showing similar results.

### Mouse hepatocytes culturing and viability assay

Primary mouse hepatocytes were isolated and cultured according to a previously published protocol^102^ with minor modifications as described below. The cells were plated in William’s Medium E medium containing 50 IU Penicillin-Streptomycin, 25 mg Glutamine/Gentamycin, 5 mg Insulin-Transferrin-Selenium [ITS], and 2 μg Glucagon (plating medium). Livers from 8 to 12 weeks C57BL/6 female mice were first perfused with PBS. The second perfusion was performed via retrograde cannulation of the inferior vena cava and egress through the portal vein using 0.48 mg/mL collagenase IV (Sigma-Aldrich, St. Louis, MO), which resulted in cell suspension that was passed through a 70 μm cell strainer. The cell suspension was then centrifuged at 50×*g* for 2 min at 4 °C after which the pellet was resuspended in a solution of plating medium and 100% Percoll (GE Healthcare Life Sciences, Piscataway, NJ), 25:12, respectively. The suspension was centrifuged again at 50×*g* and 4 °C for 10 min. The supernatant was removed and the pellet was resuspended in the plating media and passed through a 70 μm cell strainer again in order to obtain a single-cell suspension.

For survival assays, the cells were plated in 96 well plates at a density of 40 K cells/well. The cells were allowed to adhere for 6–12 h and then were subjected to the drug treatment. After 48 h of the drug treatment period, the media was replaced with fresh plating media containing MTS reagent (Promega—CellTiter 96 AQueous One Solution). The cells were incubated for 5–6 h following which absorbance values were obtained and cell viability was calculated as described above. Each point reflects at least four replicates. Each plot represents at least two independent experiments with similar results.

### BC organoids and combination treatment

The organoid lines 5079 and 4013 were derived from BC PDX tumors and obtained from Baylor College of Medicine PDX Core. The 5097 PDX line was collected from a patient who had an ER+ intraductal micropapillary carcinoma with a BRCA2 mutation and the tumor was passaged three times in SCID mice before use^103^. The 4013 PDX line was derived from a triple-negative infiltrating ductal carcinoma patient and was transplanted into mice eight times before use^103^. Organoid culture maintenance for MCF-7 cells-derived organoids, 5097 PDX organoids, and 4013 PDX organoids was performed following previously published protocol^76^. In brief, for PDX tumor tissues, the tissues were cross-cut into small pieces, minced completely, and digested with collagenase I and III, followed by filtering through a 70 µm nylon mesh cell strainer to obtain single cells. Then, for all the single-cell samples, including MCF-7 cells, the cells were suspended in a small volume of culture medium that contains R-spondin 3 and Noggin, along with additional growth factors^76^, and mixed with collagen gel at a v/v ratio of 1:4. 40 μL of the cell-collagen mixture was placed into each well of 24-well plates and solidified by incubation at 37 °C with 5% CO_2_ for 30 min. Next 1 ml of culture medium was added to each well and cells were incubated at 37 °C with 5% CO_2_ overnight.  Following the overnight incubation period the cultures were given with drugs to achieve final concentrations of: 50 nM SI-12, either alone or in combination with: 250 nM  SGI-1027, 312 nM Bay850, 156-312 nM Atuveciclib and 5 μM H89 . Organoids were continuously cultured for 2 weeks. Fresh medium and drugs were provided every 3–4 days. At the end of the treatment period, the organoids and cells were counted.

### siRNA sequences and transfection

The 27-mer Dicer-substrate siRNA (DsiRNA) duplexes (referred to as “siRNA”) were purchased from integrated DNA technologies (IDT). The sequences of siRNA oligomers are listed in Supplementary Data 1, Table S5. Cells were seeded in 150 mm dishes and allowed to adhere overnight in an appropriate full growth media at 37 °C under a 5% CO_2_ atmosphere. Following the overnight incubation period, the cells were supplied with fresh media containing siRNA/Lipofectamine RNAiMAX (ThermoFisher # 13778150) complexes. The complexes were prepared according to the manufacturer’s instructions and contained  either three targeting siRNA sequences (10 nM each) or negative control (NC) siRNA, resulting in a 30 nM final siRNA concentration, unless mentioned otherwise. The transfected cells were incubated for 48 h after which they were washed, harvested, and subjected to downstream procedures.

### Real-time quantitative polymerase chain reaction (RT-qPCR)

RNA was extracted from cells using TRIzol reagent (Invitrogen). Reverse transcription cDNA synthesis was performed with the VILO SuperScript cDNA Synthesis Kit (Invitrogen) using 2 μg of total RNA. Probe-based RT-qPCR was performed on a StepOnePlus Real-Time PCR machine (Applied Biosystems) using probes from the Universal Probe Library (Roche) and TaqMan Universal Master Mix (Applied Biosystems). Primers were designed on the “universal probe library assay design center system” platform (Roche). Relative mRNA expression levels were calculated by the ΔΔCT method with normalization to GAPDH or ACTB. Each result is represented by standard deviation (SD) of at least three technical replicates. Primer sequences and probes numbers are available in Table S6.

### Western blot

Immunoblot analysis was performed by standard procedure. In brief, proteins from whole-cell lysates were obtained by using RIPA lysis and extraction buffer (TermoFisher, cat no. 89900). Equal protein amounts per well were loaded and run on a 4–20% precast polyacrylamide gels (BioRad, cat no. 4561096). The protein samples were transferred to nitrocellulose membranes using a semi-dry transfer system (TermoFisher, iBlot Gel Transfer Device), blocked with PBS-Tween buffer containing 5% nonfat milk dissolved powder for 60 min, thoroughly washed and then incubated overnight at 4 °C with primary antibodies (c-Jun #9165, CST; HSP90 #4877, CST). After thorough washing, the blots were incubated (at room temperature, 60 min) with a secondary antibody (anti-rabbit, CST, 7074S) coupled to horseradish peroxidase. Membranes were then washed and protein bands were detected with Pico PLUS Chemiluminescent substrate (TermoFisher, cat no. 34580) in a V3 Western Workflow (BioRad).

### GeCKOv2 CRISPR-Cas9 library amplification

Two SGR GeCKOv2 sub-libraries (A and B) were obtained from Addgene (#1000000048) deposited by the Feng Zhangs' laboratory. The libraries were amplified by performing six reactions per sub-library (allowing one reaction per ~10 K plasmids). In total, 25 μL Endura ElectroCompetent cells (Lucigen #60242) were electroporated with 100 ng plasmid (2 µL of a 50 ng/µL stock) in a 0.1 cm electroporation cuvette (Bio-Rad #1652089). The cuvette was placed in the electroporator (Bio-Rad #411BR) and pulsed at 1.8 kV for a duration of 5 ms. In total, 1.975 mL recovery media was added to each cuvette and then the content of a cuvette was transferred into a 15 mL tube, which was loosely capped and allowed to shake for 1 h in an incubator at 37 °C, 250 rpm. All the six tubes (for each sub-library) were pooled. For estimating the transformation efficiency dilutions of 1/10 K, 1/50 K, and 1/100 K were plated on separate ampicillin-containing 150 mm agar plates, then the rest of the bacterial pool was plated on 150 mm ampicillin-containing agar plates (400 µL per plate). All the plates were incubated over-night at 32 °C after which colonies on the diluted plates were counted. The estimation of colony count was >6 M total colonies, which means that every SGR is represented by at least 100 colonies. All the bacterial colonies were rinsed with LB medium and gently scraped and pooled. To collect all the remaining bacteria, the plates were washed with a minimal volume of LB medium. All the bacteria containing LB media was transfered into 50 mL polypropylene centrifuge tubes, centrifuged to concentrate the bacterial pellet and after removal of the liquids, all the bacterial pellets were processed for DNA plasmid purification using the Plasmid Plus Maxi Kit (Qiagen #12963) and following the manufacturer’s instructions. The extracted plasmid libraries were pooled and quantified using a NanoDrop spectrophotometer.

### NGS for verifying preservation of library complexity

For amplifying the SGR target region the plasmid library was subjected to a first PCR cycle using Fwd: CCCTACACGACGCTCTTCCGATCTGCTTTATATATCTTGTGGAAAGGACGAAACACC and Rev: GTGACTGGAGTTCAGACGTGTGCTCTTCCGATCTCCGACTCGGTGCCACTTTTTCAA primers. Each PCR reaction mix was prepared as following: Next Ultra II Q5 PCR Master Mix*2 (NEB # M0544) 25 µL, pooled SGR library plasmid DNA template at a final concentration of 400 ng/mL, each of the two primers at a final concentration of 0.25 µM and ultra-pure water to complete the reaction to 50 µL final concentration. The PCR reaction mix was subjected to cycling conditions that are described in Table S7.

The crude PCR product was purified using a QIAquick purification kit (Qiagen #28106) following the manufacturer’s instructions. The purified PCR product was quantified using a NanoDrop spectrophometer and an aliquot equivalent to 2 µg was subjected to agarose gel separation (2%). A band at ~230 bp was extracted from the agarose gel using the QIAquick extraction kit (Qiagen # 28706).

The gel-extracted dsDNA was quantified by Qubit and 10 ng aliquots were used as templates for the second round of PCR using Illumina adaptors (P5 and P7)-containing primers Fwd: AATGATACGGCGACCACCGAGATCTACACTCTTTCCCTACACGACGCTCTTCCGATC*T and Rev: CAAGCAGAAGACGGCATACGAGATNNNNNNNGTGACTGGAGTTCAGACGTGTGCTCTTCCGATC*T (NNNNNNN: a unique index used for a distinction between different samples). For the second PCR reaction, the Q5 Hot Start High-Fidelity Mastermix (NEB # M0494) was used to amplify the pool in ten cycles. The final PCR products were column purified using DNA Clean and Concentrator (Zymo Research, D4013), loaded onto a Novaseq sequencer, and subjected to massively parallel sequencing to obtain at least 50 M reads per sample. Two independent sample sets were used for T0 and a single sample for T1, T2, and T3.

The sequencing data were analyzed by applying a previously published python script ^40^ and the results met the advised parameter specifications for library quality with a nearly ideally preserved SGR distribution (complexity).

### Lentiviral production

Twenty plates of low passage (<10) 293FT cells were plated on 15 cm dishes (1.2 × 10^7^ cells/plate to allow ~80% confluence) and allowed to adhere overnight. Lentiviruses were generated by co-transfecting either the pooled GeCKOv2 human whole-genome library (comprised of an equal contribution of the two sub-libraries—Lib.A and Lib.B) or pLJM1-EGFP (Addgene, cat. no. 19319—a gift from David Sabatini), with packaging vectors psPAX2 (Addgene, #12260) and pMD2.G (Addgene, #12259)—both are gifts from Didier Trono. The plasmid mixture was prepared as follows: psPAX2/MD2.G/GeCKOv2 (as a mix of Lib.A + Lib.B) or pLJM1-EGFP 17.5/9.5/25 or 18 μg, respectively, were mixed and 150 µL transfection reagent (XtremeGENE9 DNA—Roche, Sigma Millipore # 6365787001) was diluted in 5 mL OptiMEM medium and incubated for 5 min at room temperature. Then the plasmid mixture was added into the transfection reagent containing OptiMEM media. The plasmid mixture was mixed and incubated with the diluted transfection reagent for 30 min at room temperature, after which the mixture was added dropwise into the 293FT culture dish.

The infected cultures were incubated overnight, after which the media was removed, and cells were provided with 16 mL/plate antibiotic-free fresh FBS containing media supplied with 1% pre-sterilized BSA. During the following 48 h, the virus-containing media was collected twice (each 24 h) from all the GeCKOv2 transfected cell culture dishes, filtered (0.22 μm), and pooled. The pLJM1-EGFP transfected cells were inspected under a fluorescent microscope and the virus was collected separately in the same manner. During the viral supernatant collection period (48 h), the pooled supernatant was kept on ice and then divided into 10 mL portions and finaly stored at −80 °C.

### MOI determination

MCF-7 cells were plated on 15 cm plates in two groups (8–10 × 10^6^ cells/plate, without antibiotics) along with the viral supernatants at various concentrations as described in Table S4.

Twenty-four hours after the viral infection, the media was removed from the cultures, and cells in group 1 were provided with fresh media containing 2.5 μg/mL puromycin (pre-determined as minimal 100% lethal concentration at 72 h exposure). Cells in group 2 were provided with fresh media without puromycin. Puromycin selection took place for 72 h, after which no living cells were observed in dishes 1 and 2 of group 1. The amount of cells in each individual dish was counted. The amount of cells in dishes 1 and 2 of group 2 was presumably the same (about 3 × 10^7^ cells), indicating that there was no polybrene-related toxicity. MOI was calculated by dividing the amount of cells in a dish from group 1 by the amount of cells in a corresponding dish (with a similar number) in group 2, revealing that 4 mL virus in 16 mL culture media results in MOI 0.25–0.3.

### Screening

In total, 10^9^ MCF-7 cells were seeded in 15 cm culture dishes (10^7^ cells/dish—100 dishes in total) along with polybrene (8 µg/mL) and 4 mL of virus solution/dish. Twenty-four hours after infection the viral media was removed, and the cells were provided with fresh media containing 2.5 μg/mL puromycin. Seventy-two hours after puromycin was added, the media was removed from the cultures, and the cells were washed, trypsinized, and pooled. At this point, three fractions of 70 million cells each were collected, pelleted, and stored at −20 °C to allow gDNA harvesting (T0). The rest of the cells were divided into three groups, plated again in 15 cm dishes (10^7^ cells/dish), and allowed to adhere overnight, then the old media was removed and the cultures were provided with fresh media containing either DMSO (group 1), SI-12 (group 2) or ICI (group 3). Each one of the three groups was divided into three arms to allow biological replicates and the following treatment regimen was applied: the starting concentration of both drugs, SI-12 and ICI, was 10 nM. During the course of the treatment period, drug concentration was gradually increased, according to the timeline described in Fig. 1c. At each time-point, all the cells belonging to the same arm were pooled and counted. To allow >500× library coverage, 70 million cells per arm were pelleted and stored at −20 °C for gDNA harvesting. The rest of the cells were re-plated, allowed to adhere, and subjected to continuing compound/vehicle treatment.

### gDNA extraction and preparation for NGS

gDNA was harvested from the pelleted cell samples with the Blood & Tissue Kit (Qiagen #69506) following the manufacturer’s protocol. Purified harvested gDNA was quantified to assure a sufficient amount of gDNA to maintain coverage of >500 reads per SGR and re-extraction of a left-over gDNA from the column was applied if needed (to yield >350 μg gDNA per sample). All the extracted gDNA was subjected to two rounds of PCR amplification, purification, and NGS of the SGR bar-coded regions for each SGR as described above (“NGS for verifying preservation of library complexity” section).

### Data analysis of SGR read counts

To retrieve the actual SGR counts from the FASTQ files we used a previously published count_spacer.py script^40^. The normalized values for each SGR were obtained as follows: $N_{sgRNA} = {\mathrm{normalized}}\,{\mathrm{SGR}} = \frac{{{\mathrm{actual}}\,{\mathrm{count}}\,{\mathrm{of}}\,{\mathrm{a}}\,{\mathrm{SGR}}}}{{{\mathrm{total}}\,{\mathrm{number}}\,{\mathrm{of}}\,{\mathrm{reads}}\,{\mathrm{within}}\,{\mathrm{the}}\,{\mathrm{sample}}}} \cdot 10^7$ (for a summary of the total number of reads for each sample see Supplementary Data 3). All the reads that at T0 achieved less than ~50 reads (~10% of the expected counts) were considered as noisy. All the non-noisy SGRs were used for the formation of terrace and DRACO ranking plots. For the terrace plots, we first computed the log-fold change ($L_{FC}$) values, with respect to the control of the same time point, for each $N_{sgRNA}$ pair (in drug and control samples) as follows: $L_{FC} = {\mathrm{log}}_2\left( {\frac{{{\mathrm{N}}_{{\mathrm{SGR}}}\left( {{\mathrm{drug}}} \right) + 1}}{{{\mathrm{N}}_{{\mathrm{SGR}}}\left( {{\mathrm{control}}} \right) + 1}}} \right)$. Then we calculated the mean value for all the $L_{FC}$ values ($\mu _{L_{FC}}$) that have the same gene target. Subsequently, we ranked the genes on the terraced plot according to the number of $L_{FC}$ values that have the same sign (+ or −) as the corresponding $\mu _{L_{FC}}$.

For the DRACO ranking plots, we used all the non-noisy reads and applied additional filtration that removed all the genes for which less than four $L_{FC}$ had the same sign as the $\mu _{L_{FC}}$. For each of the passed genes, we selected the SGR with the highest absolute $L_{FC}$ value (the most efficient SGR). Then, we plotted all the most efficient SGRs according to their actual (not absolute) values on a 2D grid.

### Statistics and reproducibility

For all the drug cytotoxicity assays and qPCR experiments, statistical analysis was determined by a two-tailed Student’s *t*-test. The number of technical replicates and independent experiments that were performed is indicated in the figure legends and/or individual sections within methods when applicable. Data were considered statistically significant when *p*-value < 0.05. Statistical analyses were performed with GraphPad Prism 8 (GraphPad Software, Inc., San Diego, CA). Correlation analyses were performed using the Pearson correlation coefficient.

### Reporting summary

Further information on research design is available in the Nature Research Reporting Summary linked to this article.

## Supplementary information

Supplementary Information

Description of Additional Supplementary Files

Supplementary Data 1

Supplementary Data 2

Supplementary Data 3

Reporting Summary

## Data Availability

NGS data are available in the NCBI Sequence Read Archive (BioSample accession: SAMN17137021). Count reads supporting all the terrace and DRACO plots, as well as data that supports the analysis presented in Figs. 2e and 8b are all available in Supplementary Data 1. Source data underlining all the other graphs presented in the main body of the manuscript is available in Supplementary Data 2. The summary of a number of processed reads in SI-12 and ICI screens is shown in Supplementary Data 3. All the other raw data-sets are available from the corresponding author upon request.

## Code availability

The github repository that contains the Python Jupyter notebooks used to analyze the results and generate the terrace and DRACO plots is available at: https://github.com/BCM-CRISPR/BCM-CRISPR.github.io In addition, we offer an interactive website to enhance the exploration of the data in the GitHub repository via: https://bcm-crispr.github.io/
