# Supplementary material for: A genome-scale CRISPR Cas9 dropout screen identifies synthetically lethal targets in SRC-3 inhibited cancer cells
Source: Commun Biol. 2021 Mar 25;4:399. doi: 10.1038/s42003-021-01929-1 (PMC7994904; doi:10.1038/s42003-021-01929-1)
Supplement: Supplementary file 1 — Supplementary Information [file 42003_2021_1929_MOESM1_ESM.pdf]

Supplementary information

A Genome-Scale CRISPR Cas9 Dropout Screen Identifies Synthetically Lethal Targets in SRC-3 Inhibited Cancer Cells

Yosi Gilad[1], Yossi Eliaz[2], Yang Yu[1], Adam M. Dean[1], San Jung Han[1], Li Qin[1], Bert W. O'Malley[1]* and David M. Lonard[1]*

[1]Department of Molecular and Cellular Biology, Baylor College of Medicine, Houston, Tx 77030, USA [2]Department of Molecular and Human Genetics, Baylor College of Medicine, Houston, TX 77030, USA

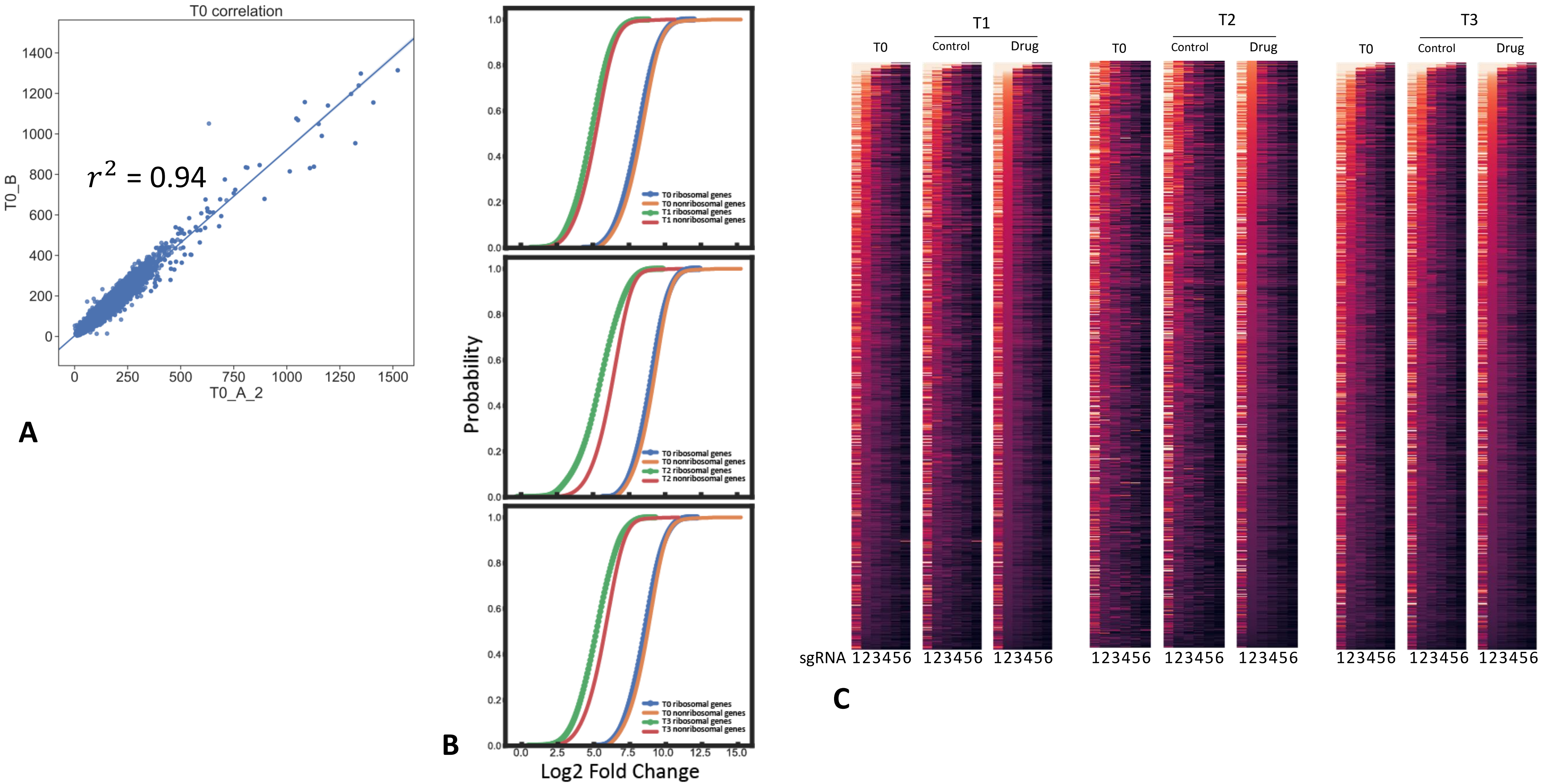

Figure S1. Properties of the experimental setup. **a.** Pairwise correlation between time point T0 replicates. **b.** Cumulative distribution fraction (cdf) scores of ribosomal vs non-ribosomal genes. **c.** sgRNAs within the same set (six sgRNAs that target the same gene) generally possess different gene-editing efficiency. The heatmaps represent time points T1, T2 and T3. The sorting was done according to the 2nd highest counted ('2nd best') sgRNA in every set in the 'Drug' sample of each time point.

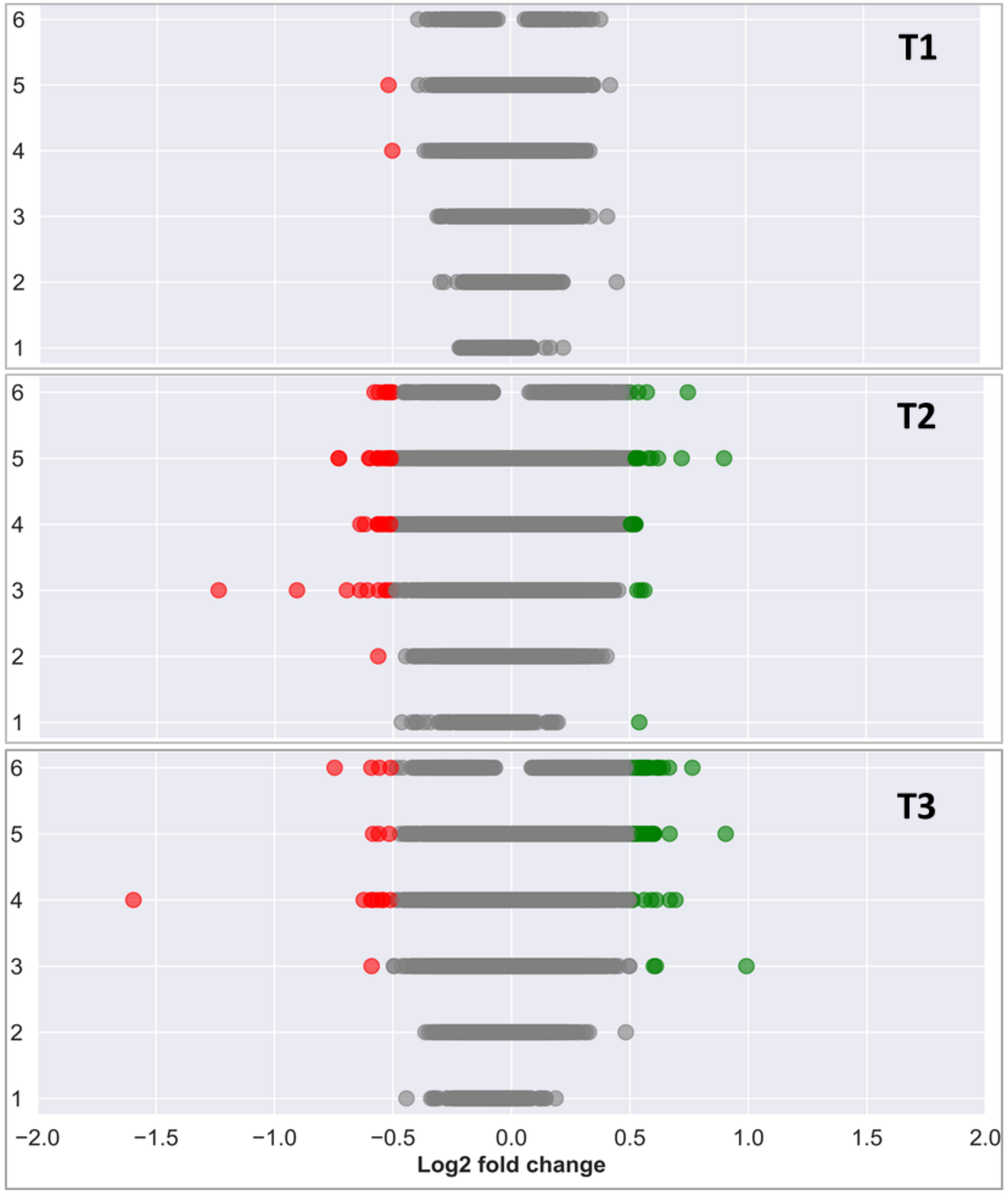

Figure S2. Terrace plots of time points T1-T3 show that genetic dependencies of the cells for survival increase proportionally to the intensity and duration of pharmacological pressure

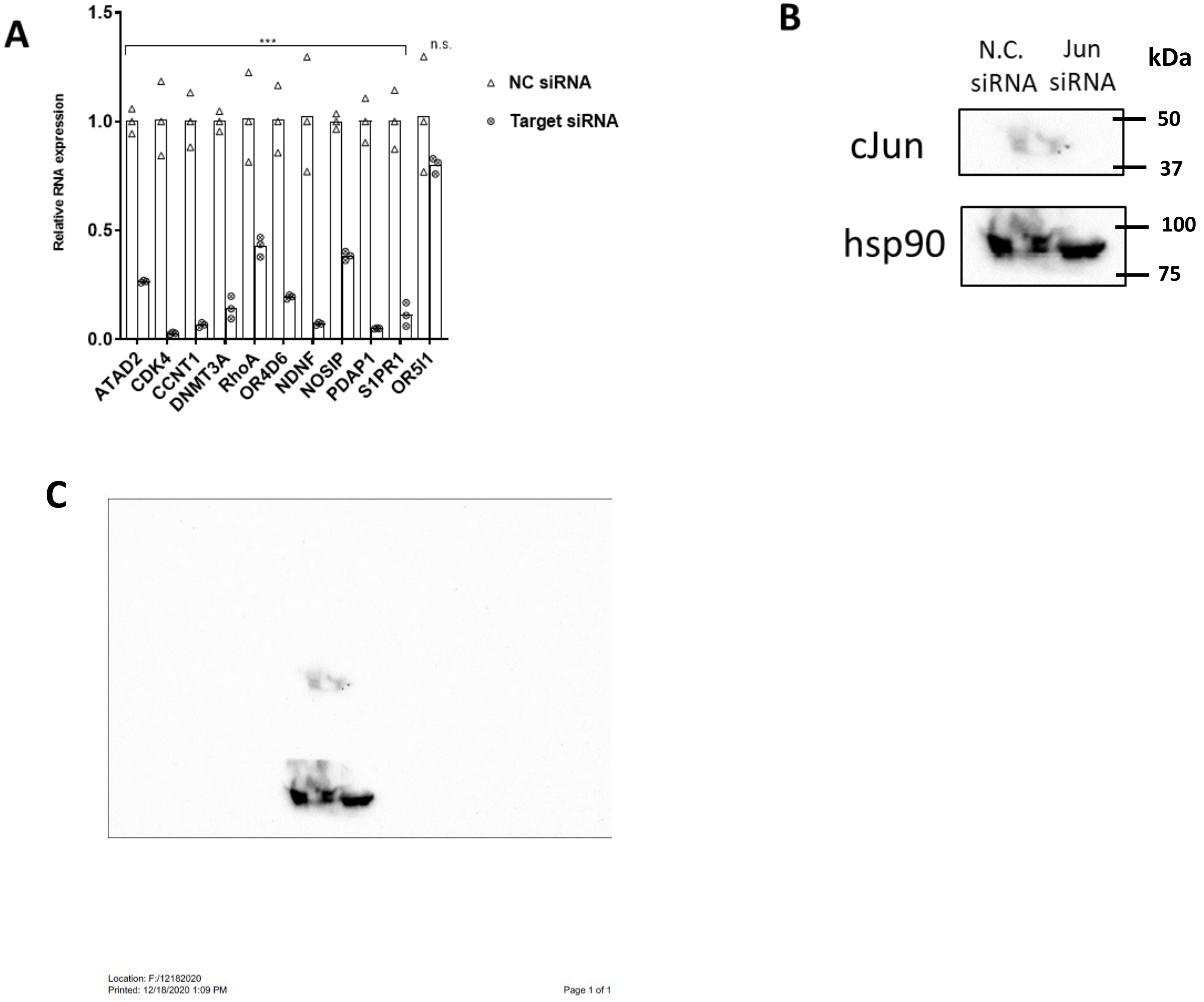

Figure S3. MCF-7 cells were treated with 10 nM of the indicated target siRNA or negative control siRNA (NC) for 48 h. Following the siRNA treatment, KD efficiency was determined by either qPCR (a) or WB(b). Uncropped image of the blot from Figure S3b (c)

***P < 0.001, two-tailed Student's t-test. *n.s.*, not significant

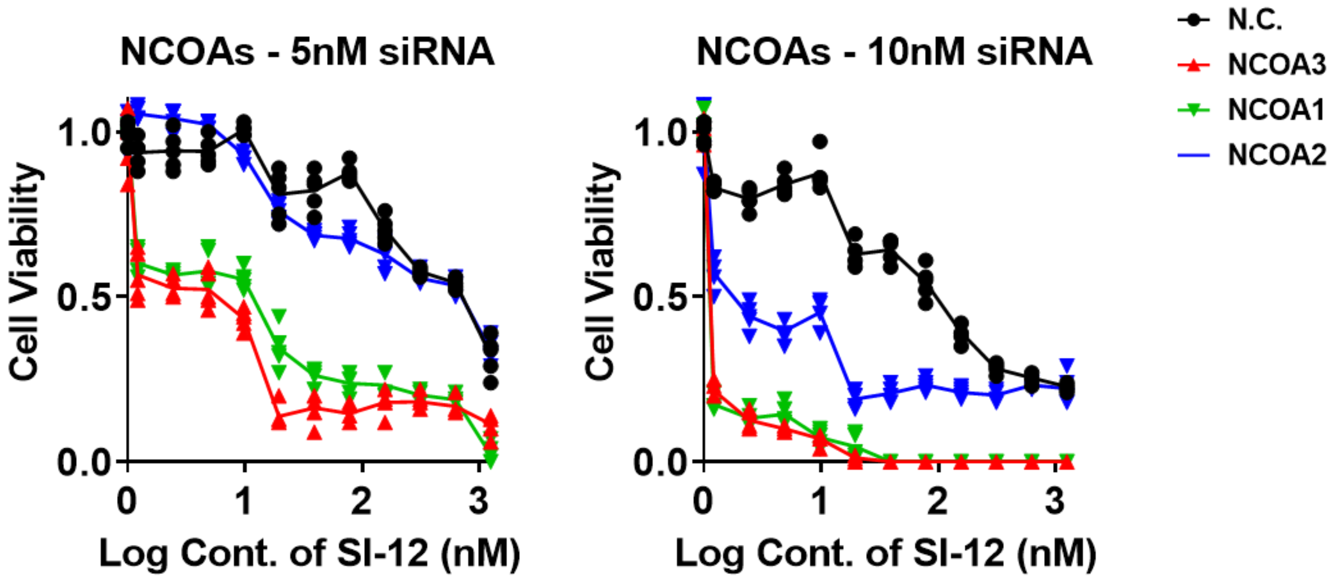

Figure S4A. siRNA gene KD experiments for NCoAs 1-3.
Cells were treated with 5 or 10 nM of the indicated siRNA for 48 h, plated in 96 well plates and exposed to SI-12 for 96 h. At the end of SI-12 treatment period, the cells were subjected to MTS viability assay.

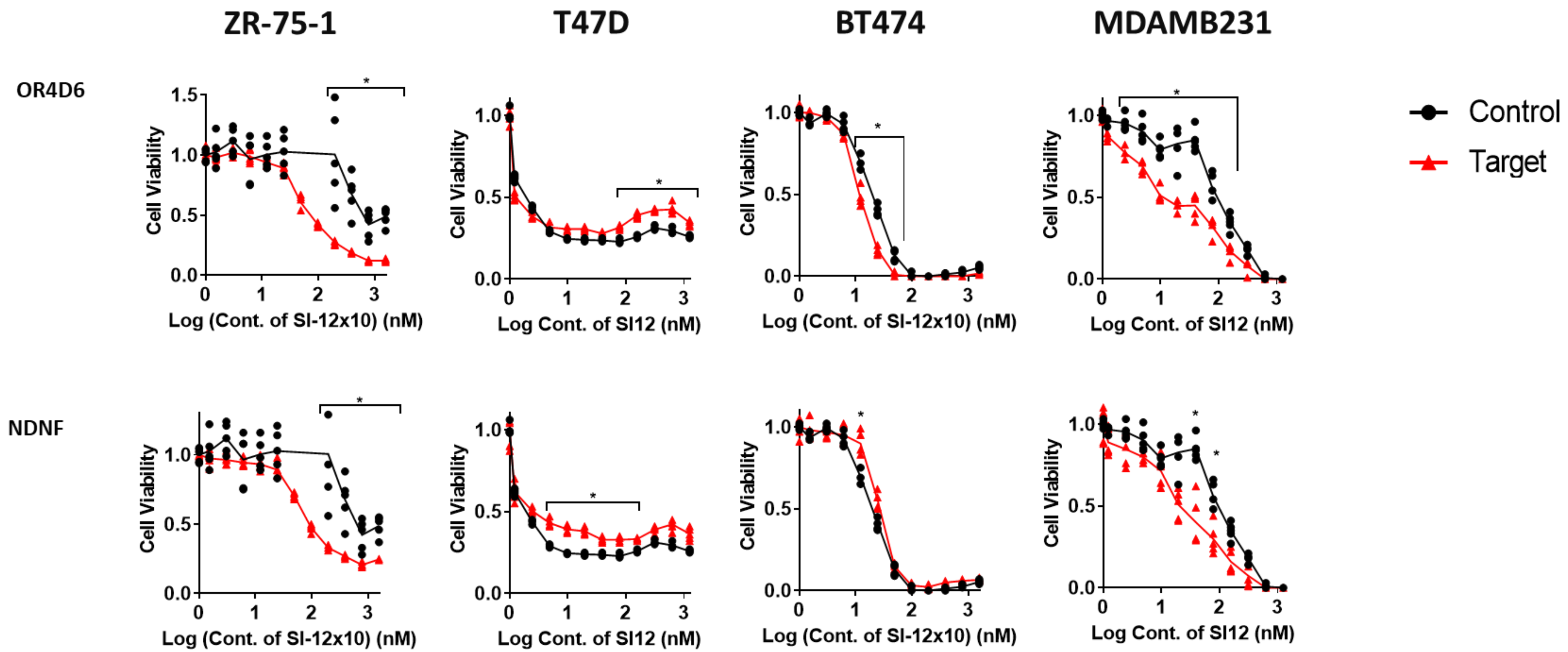

Figure S4B. Assessment of OR4D6 and NDNF as potential targets for effective combo with SI-12 by siRNA gene perturbation in BC cell lines. Cells were treated with 10 nM of the indicated siRNA for 48 h, plated in 96 well plates and exposed to SI-12 for 96 h. At the end of SI-12 treatment period, the cells were subjected to MTS viability assay. * Where mentioned *P*<0.01, two-tailed Student's t-test; if not mentioned, not significant.

Target	Available inhibitor
RHOA	CGG1423
CDK4	Abemaciclib
CCNT1	Atuveciclib (CDK9)
MAPK8 & JUN	JNK-IN-8
ATAD2	BAY-850
DNMT3A	SGI1027 (pan-DNMTi)
S1PR1	Fingolimod
NUAK2	WZ4003

Table S1. Genes from Table S1 (top 100) for which there small molecule inhibitors are commercially available. Atuveciclib – a CDK9 inhibitor - is used to target the partnership of CCNT1 with CDK9 in P-TEFb complex.

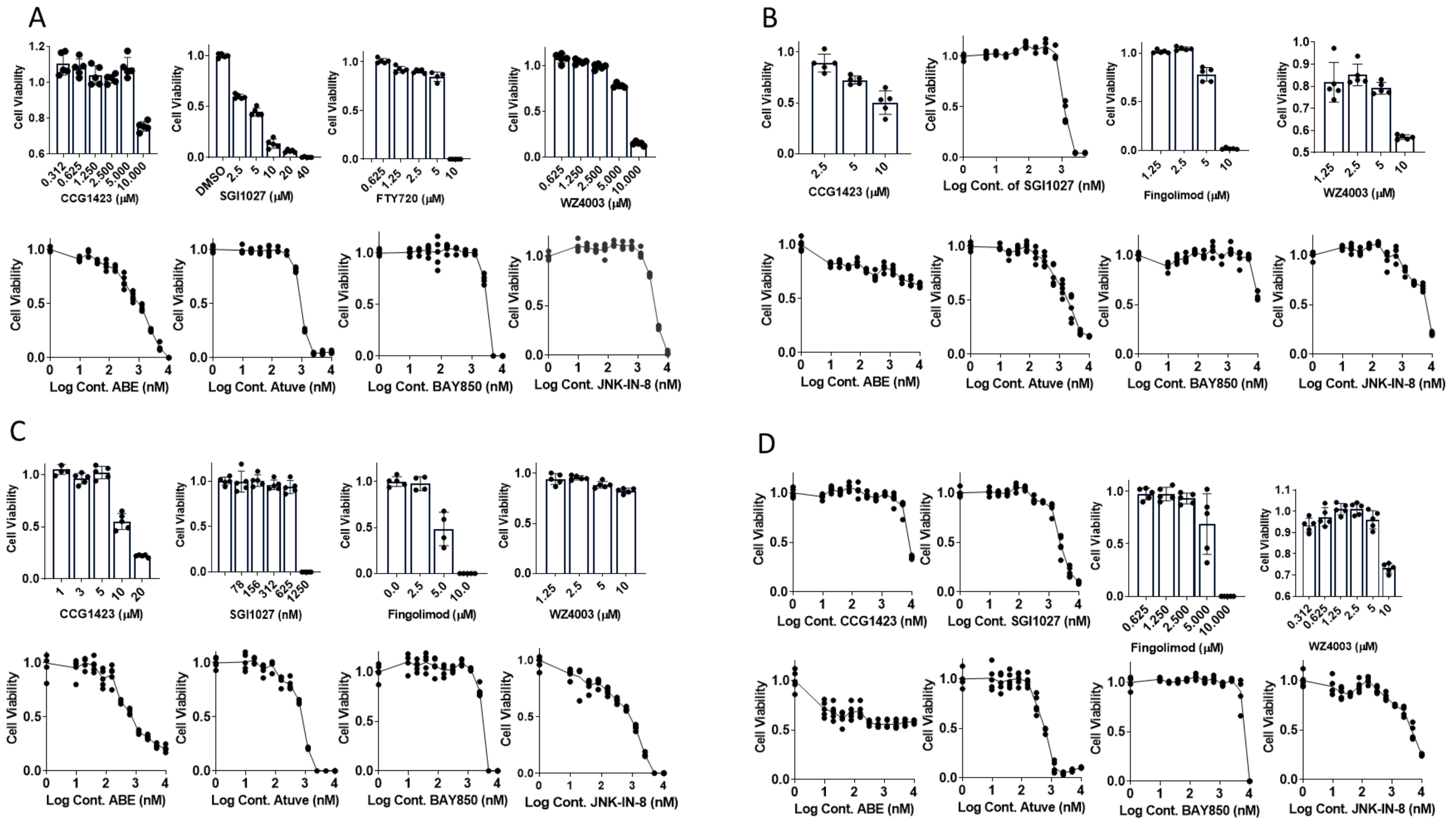

Figure S5. Assessment of bioactive concentrations of single agents. The cells were plated in 96 well plates and subsequently exposed to the compounds at indicated concentrations for 96 h, after which cell viability was assessed by MTS assay. **a.** In MCF-7 cells. **b.** In T47D cells **c.** In BT474 cells. **d.** In ZR-75-1 cells.

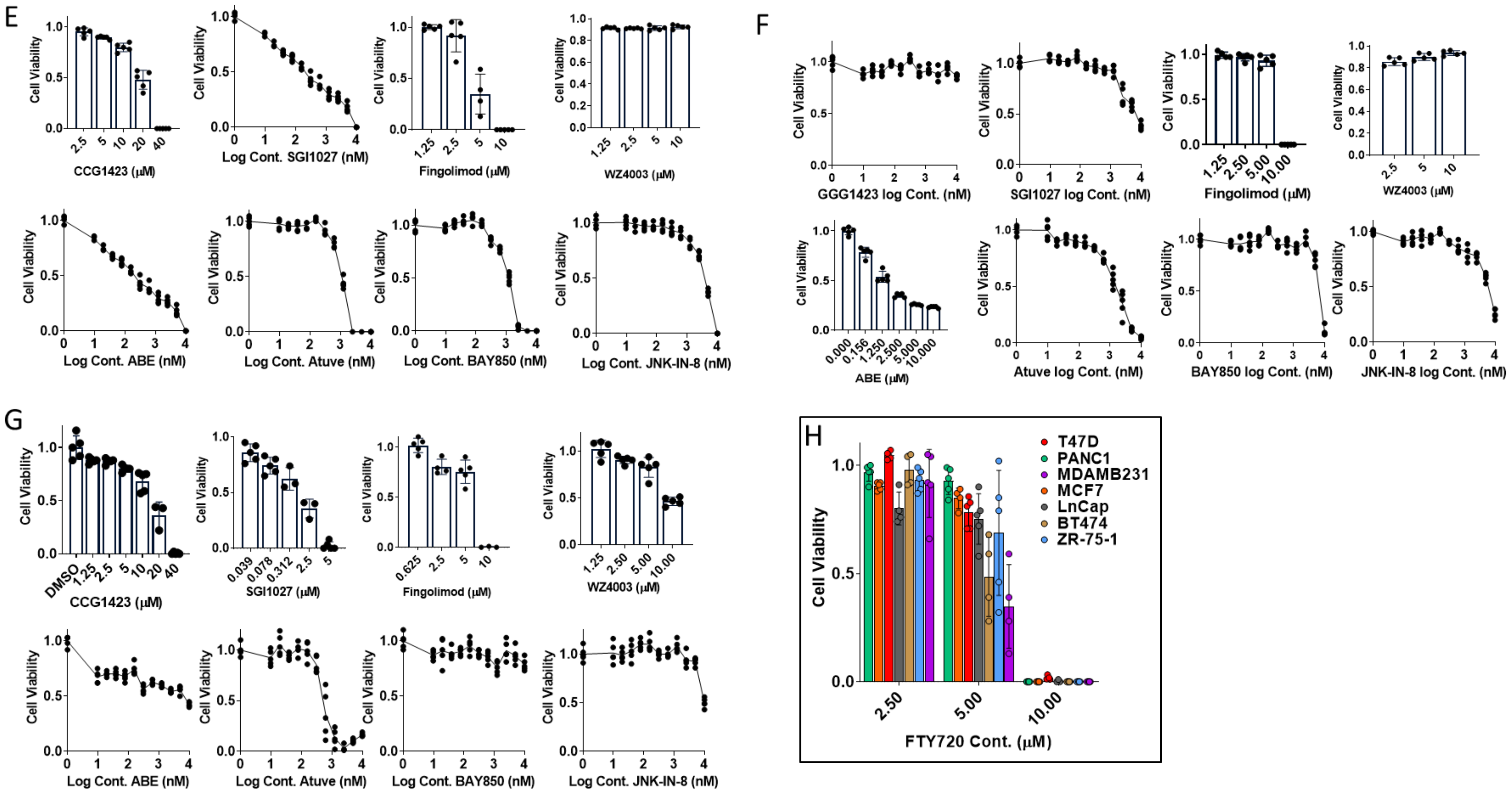

Figure S5 (continued). e. In MDAMB-231 cells. **f.** In PANC-1 cells. **g.** In LnCAP cells. **h.** Summary of fingolimod activity shows anti proliferative effect when the drug applied at high dosage across all the tested cancer cell lines, which suggests to considering its repurposing for cancer treatment.

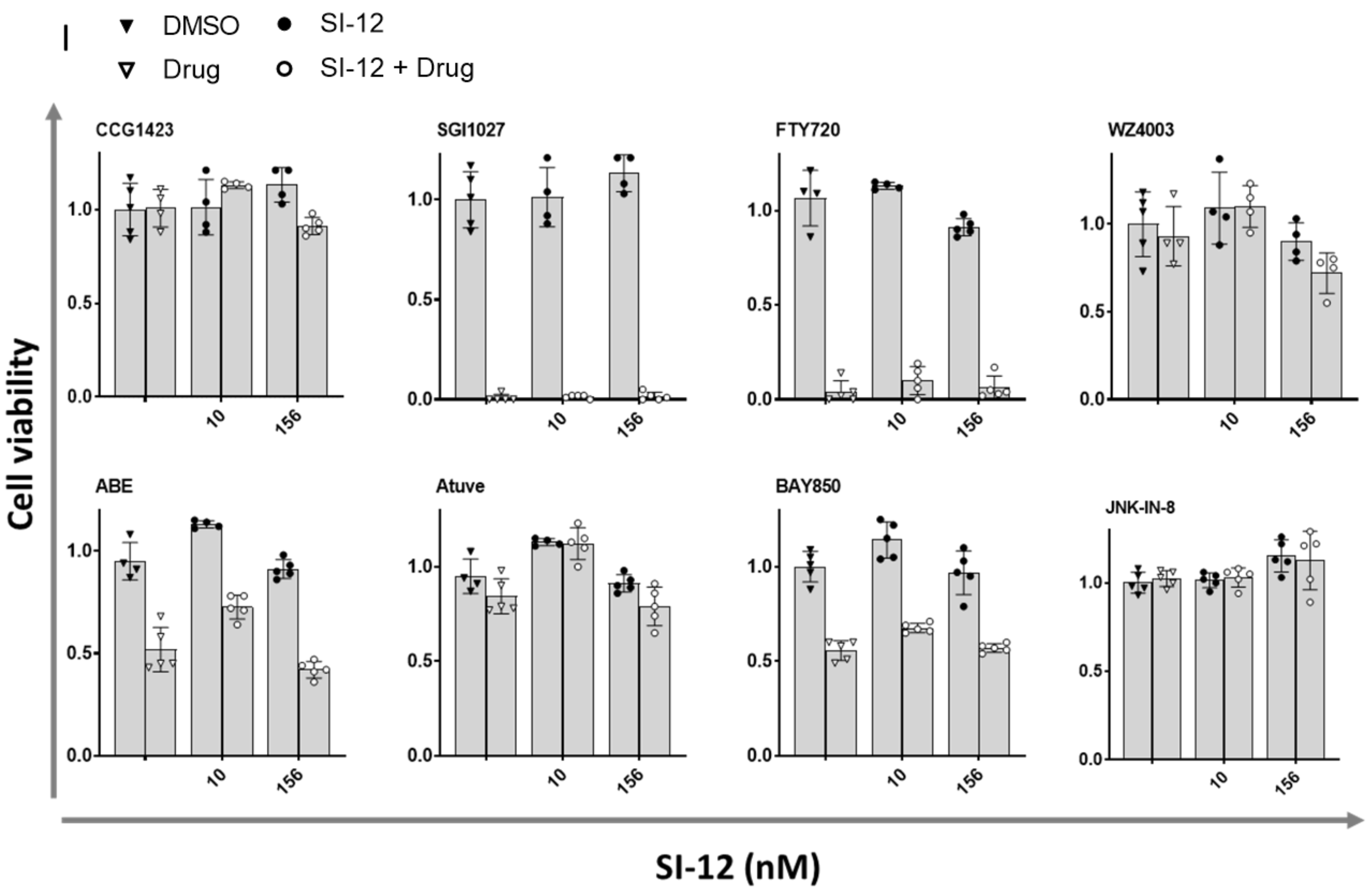

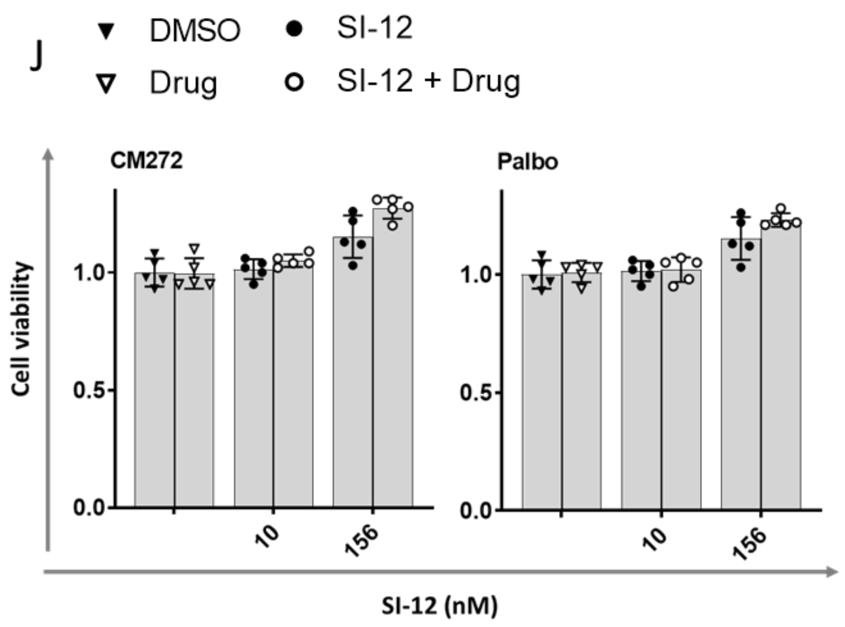

Figure S5. Viability assay of primary mouse hepatocytes treated with single compounds or with the indicated combinations. I. First set of inhibitors. **J.** Alternative DNMT (CN272) and CDK4 (Palbociclib) inhibitors.
The cells were plated in 96 well plates, treated with the compounds at indicated concentrations (**Table S2**) for 48 h, followed by MTS cell viability assay. Each point reflects at least four replicates. Each plot represents at least 2 independent experiments with similar results.

Drug	Concentration (µM)
CCG1423	10
SGI1027	5
FTY720	5
WZ4003	10
ABE	2.5
Atuve	1.25
BAY850	5
JNK-IN-8	5
CM272	0.01
Palbociclib	0.625

Table S2. Drug concentrations used for hepatocytes viability assays (Figure S5).

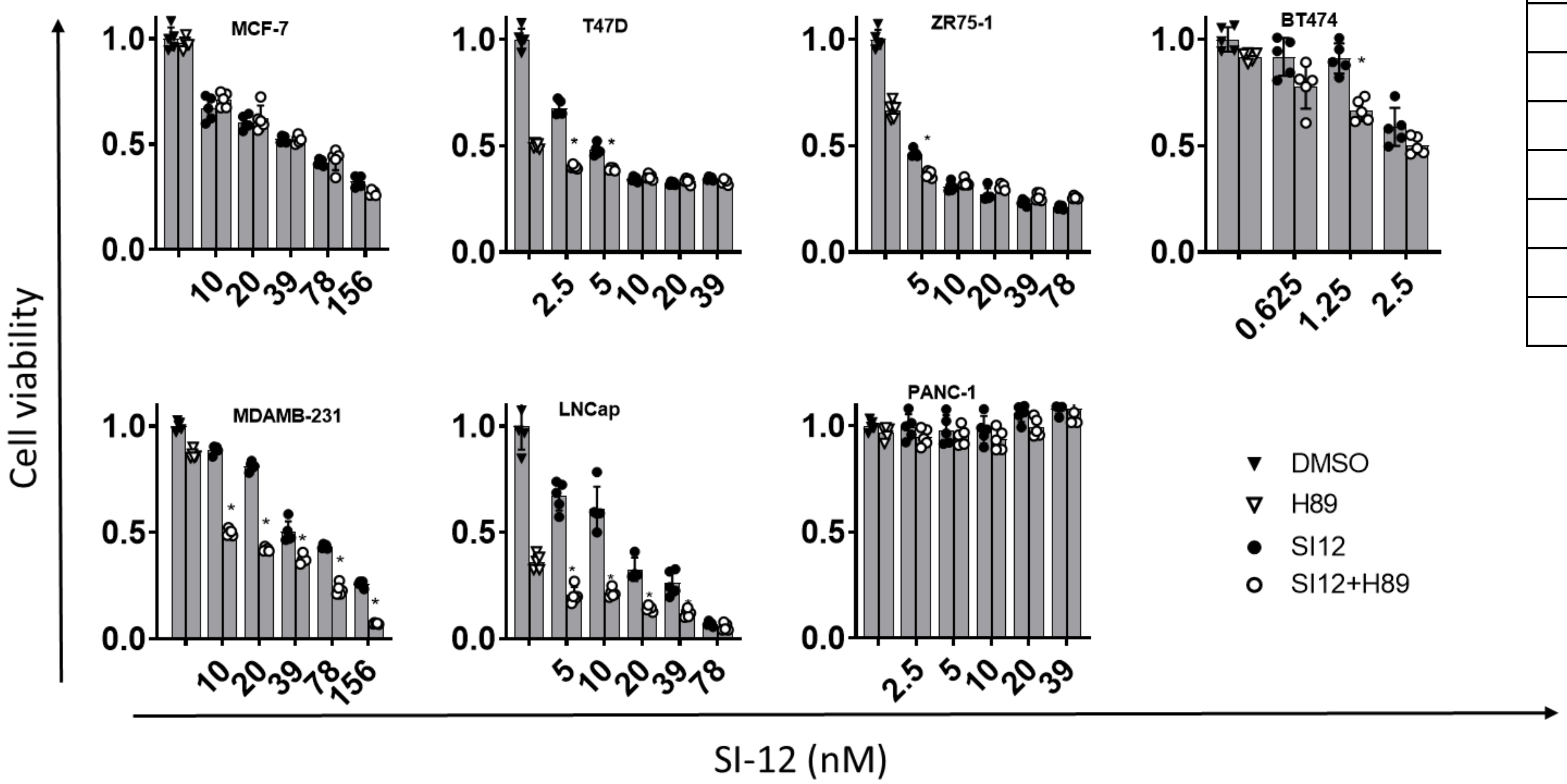

Cell line	H-89 concentration (μM)
MCF-7	2.5
T47D	10
ZR75-1	10
BT474	10
MDAMB-231	5
LNCap	10
PANC-1	5

Table S3. H-89 concentrations in Figure S5k.

Figure S5K. Cells were treated with SI-12 or H89 or the combination of SI-12 and H-89 for 96 h (For H-89 concentrations, see **Table S3**). At the end of drug treatment period, the cells were subjected to MTS viability assay.
Statistical significance compares between the combo and the most effective single agent (either SI-12 at relevant concentration, or the partner molecule). * P<0.001, two-tailed Student's t-test.

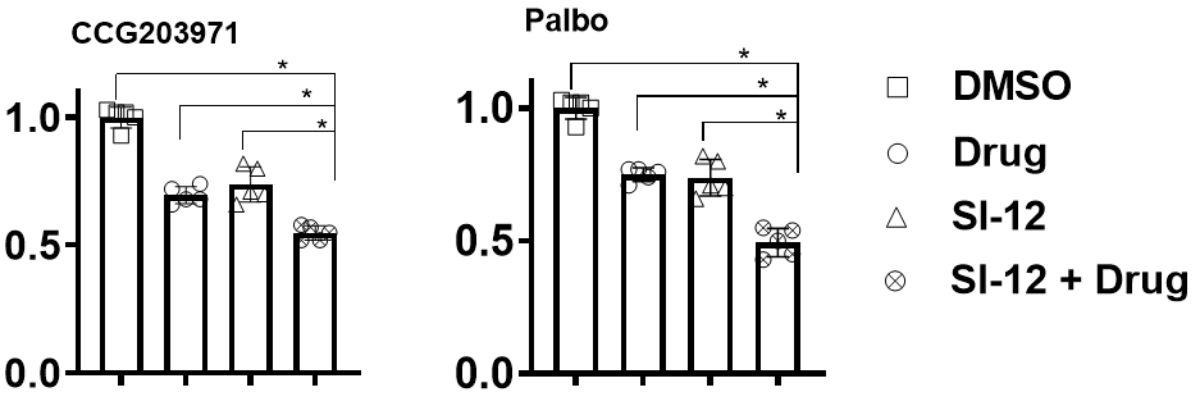

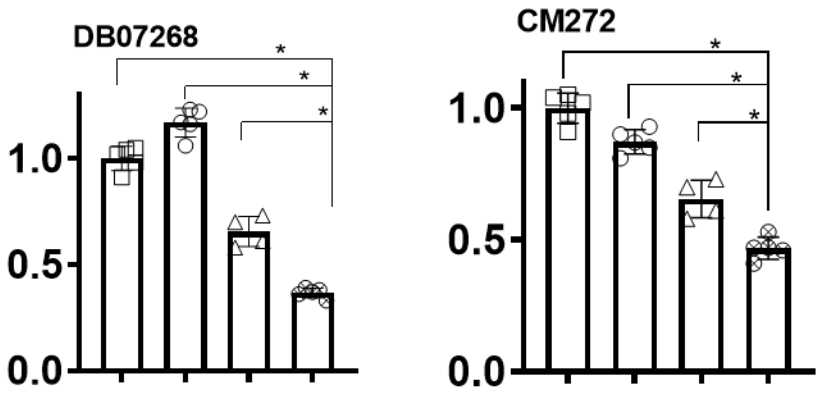

Drug	Concentration (µM)
CCG203971	10
Palbo	0.625
DB07268	10
CM272	0.01
SI-12	0.01

Figure S6. Four alternative small molecule inhibitors that target the same screen hits (Figure 3c) were tested in combination with SI-12. MCF-7 cells were plated in 96 well plates and treated with the indicated compound(s) for 96 h. At the end of drug treatment period, the cells were subjected to MTS viability assay. * P<0.001, two-tailed Student's t-test. Each point reflects at least four replicates. Each plot represents at least 2 independent experiments with similar results.

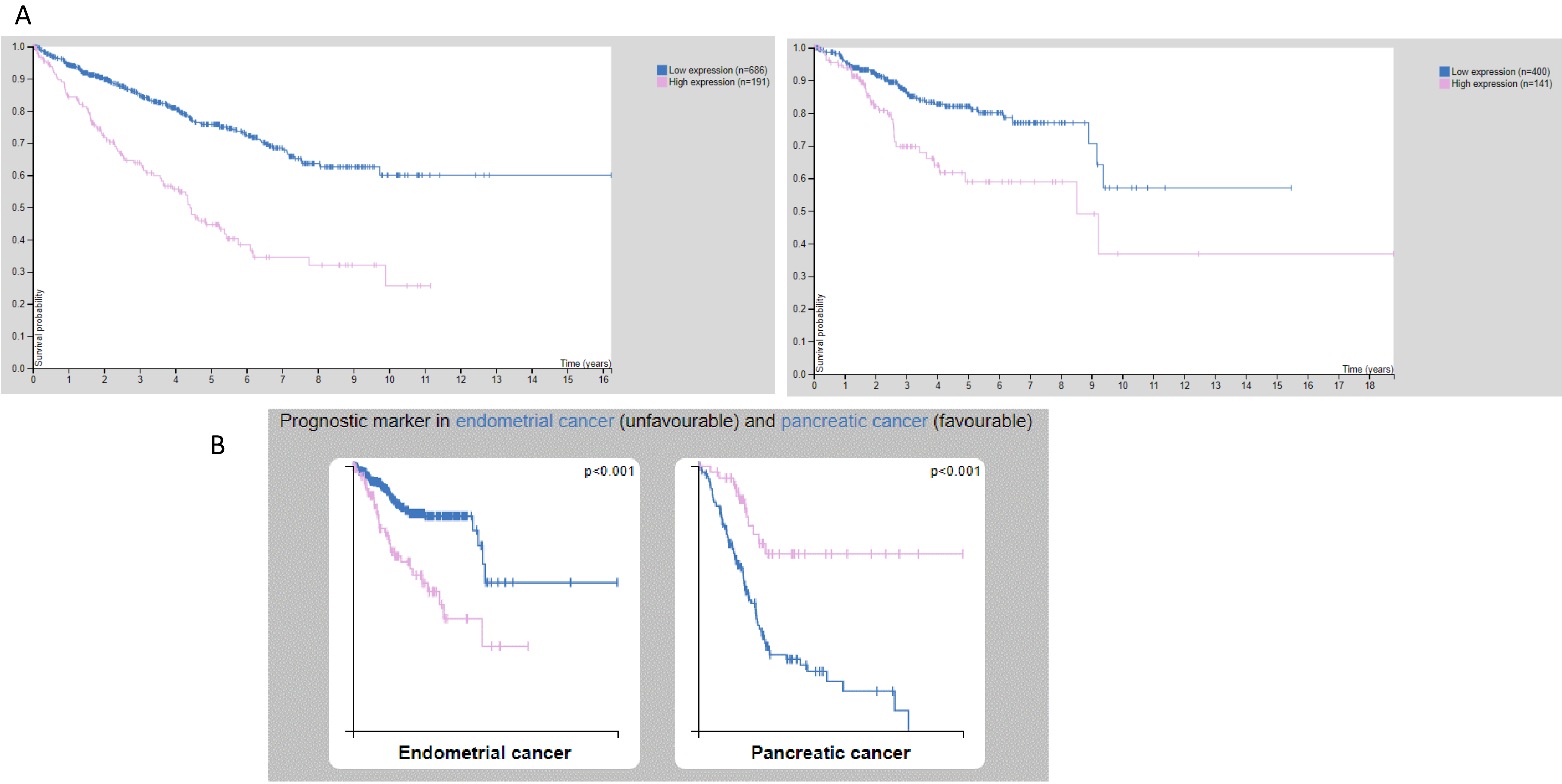

Figure S7. ATAD2 and NUAK2 prognostic analyses using data taken from The human protein atlas: **a.** The oncogene ATAD2 is unfavorable prognostic marker in renal (left) and endometrial (right) cancers. **b.** NUAK2 is favorable prognostic marker in pancreatic cancer.

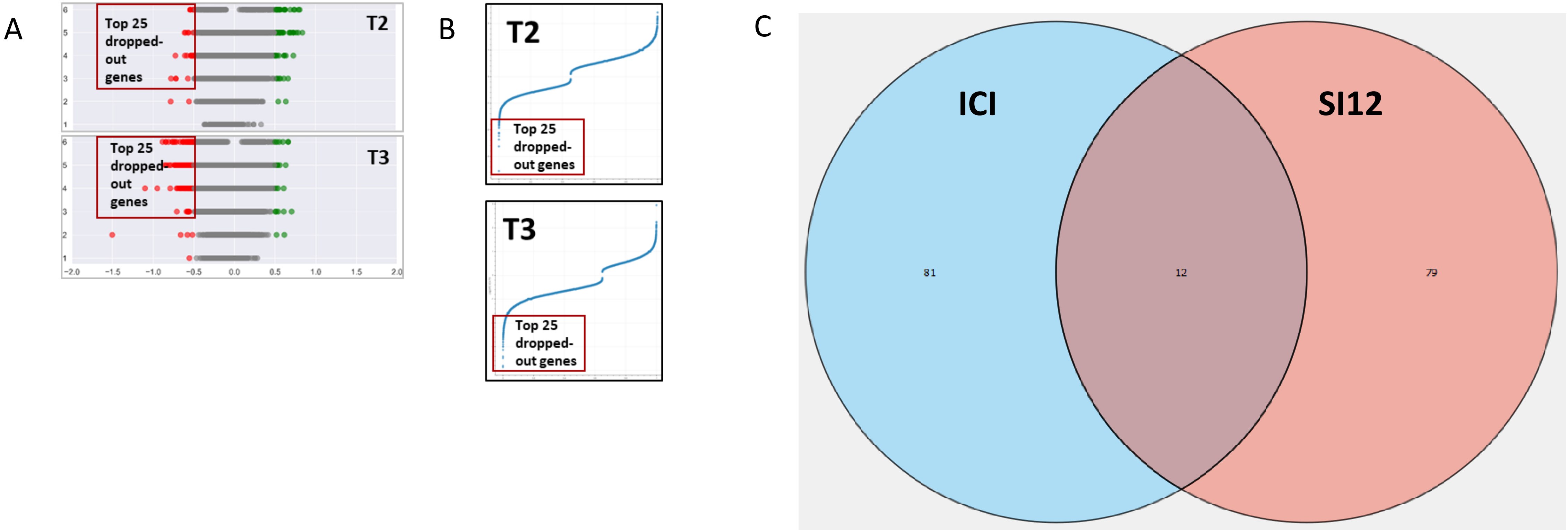

Figure S8. Comparison between ICI and SI-12 drop-out screens: Selection of top dropped-out genes in the ICI screen using Terrace plot **(a)** and Draco-based ranking **(b)**. **c.** Comparison of top 100 dropped-out genes from each screen (ICI and SI-12) reveals that 12 genes are common to both screens (*ATAD2, C17orf58, CDK4, CLPX, CTH, DEGS2, DHX36, FAM92A1, NDUFAF5, RIC8A, SLC28A2, SNRPF*) - as show in the Venn diagram.

		Group 1	Group 2
**1**	Cells only	+Puromycin	No puromycin
**2**	Cells with polybrene*	+Puromycin	No puromycin
**3**	Cells+polybrene+virus (0.25ml virus/16ml)	+Puromycin	No puromycin
**4**	Cells+polybrene+virus (0.5ml virus/16ml)	+Puromycin	No puromycin
**5**	Cells+polybrene+virus (1ml virus/16ml)	+Puromycin	No puromycin
**6**	Cells+polybrene+virus (1.5ml virus/16ml)	+Puromycin	No puromycin
**7**	Cells+polybrene+virus (2ml virus/16ml)	+Puromycin	No puromycin
**8**	Cells+polybrene+virus (3ml virus/16ml)	+Puromycin	No puromycin
**9**	Cells+polybrene+virus (4ml virus/16ml)	+Puromycin	No puromycin

Table S4. Viral titration for MOI determination. *8 µg/ml

Gene Name	Fwd. primer	Rev. primer	Probe number
ACTB	TCC CCC AAC TTG AGA TGT ATG	ACTGGTCTCAAGTCAGTGTACAGG	71
GAPDH	TCC ACC CAT GGC AAA TTC	TCCACC CAT GGC AAA TTC	9
RHOA	TAGTGGATGAGCTGTGAGTGC	AGGGTAGCGCGAGAGAGC	70
DNMT3A	ACTACATCAGCAAGCGCAAG	CACAGCATTCATTCCTGCAA	75
ATAD2	CCATGCATACTTGCTTTTGG	CCCCTCAAAAGGTTTCCAAC	7
OR5I1	TCATTCTTCTCTCAGTCTTAAAGATCC	GAGGCGCATGTAGAAAAGGT	69
OR4D6	TCCTGGGCTCCTACACTGTC	TGAAGAGTCACCACCAGCAT	9
S1RP1	CCGCCTCTTCCTGCTAATC	GCAGTTCCAGCCCATGATAG	3
NDNF	GTCAAAACCTGCAGAAAGCA	CATCCAGCAGGTAAGATTTGC	41
PDAP1	GTTCCGGTGTCAGTTCGAG	TTTCTTCCTCCTTTAGGCATTG	70
CDK4	GAGGAGTCGGGAGCACAG	CGGATTACCTTCATCCTTCTGTAGA	13
CCNT1	GAACTTTCTTATCGCCAGCAG	AGCAGTGTTGATAGTCAATTGTGAG	43
NOSIP	TACGTGTGTGCCGTGACC	ATCAGCTTCTCCACGCATTC	45

Table S6 – primers and probes (from Roche universal probe library) used for qPCR

Cycle number	Denaturation	Annealing	Extension
**1**	98°C, 3 min		
**2-23**	98°C, 10 s	63°C, 10 s	72°C, 25 s
**24**			72°C, 2 s

Table S7. PCR cycling conditions.
