# Supplementary material for: A genome-scale CRISPR Cas9 dropout screen identifies synthetically lethal targets in SRC-3 inhibited cancer cells
Source: Commun Biol. 2021 Mar 25;4:399. doi: 10.1038/s42003-021-01929-1 (PMC7994904; doi:10.1038/s42003-021-01929-1)
Supplement: Supplementary file 2 — Description of Additional Supplementary Files [file 42003_2021_1929_MOESM2_ESM.pdf]

Description of Additional Supplementary Files

File Name: Supplementary Data 1

Description:

•Comparison between the top mutated/amplified BC genes in SI-12 vs ICI screens (Fig. 8b).
•Summary of top 100 dropped out genes from the SI-12 screen (Fig. 2e).
•Top mutated and amplified genes in BC (data from TCGA).
•Sequences of commercial siRNA.

File Name: Supplementary Data 2

Description:

•Data underlying terrace plots from SI-12 screen (Fig. 2b).
•Data underlying DRACO plots from SI-12 screen (Fig. 2d).
•Data underlying olfactory receptors only terrace plots (Fig. 2f).
•Data underlying terrace plots from ICI screen (Fig. 8a).
•Data underlying cell viability experiments following siRNA perturbations (Fig. 3a, Fig. 3b, Fig. 3c)
•Data underlying cell viability experiments following drug treatments (Fig. 3d, Fig. 5, Fig. 6)•Data underlying organoid experiments (Fig 7).

File Name: Supplementary Data 3

Description:

•Summary of number of processed reads in SI-12 and ICI screens at all time points.
