# Supplementary material for: A genome-scale CRISPR Cas9 dropout screen identifies synthetically lethal targets in SRC-3 inhibited cancer cells
Source: Commun Biol. 2021 Mar 25;4:399. doi: 10.1038/s42003-021-01929-1 (PMC7994904; doi:10.1038/s42003-021-01929-1)
Supplement: Supplementary file 6 — Reporting Summary [file 42003_2021_1929_MOESM6_ESM.pdf]

nature research

Corresponding author(s): David M. Lonard

Last updated by author(s): Feb 4, 2021

Reporting Summary

Nature Research wishes to improve the reproducibility of the work that we publish. This form provides structure for consistency and transparency in reporting. For further information on Nature Research policies, see our Editorial Policies and the Editorial Policy Checklist.

Statistics

For all statistical analyses, confirm that the following items are present in the figure legend, table legend, main text, or Methods section.

n/a	Confirmed	
	☒	The exact sample size (*n*) for each experimental group/condition, given as a discrete number and unit of measurement
	☒	A statement on whether measurements were taken from distinct samples or whether the same sample was measured repeatedly
	☒	The statistical test(s) used AND whether they are one- or two-sided<br>*Only common tests should be described solely by name; describe more complex techniques in the Methods section.*
☒		A description of all covariates tested
	☒	A description of any assumptions or corrections, such as tests of normality and adjustment for multiple comparisons
	☒	A full description of the statistical parameters including central tendency (e.g. means) or other basic estimates (e.g. regression coefficient) AND variation (e.g. standard deviation) or associated estimates of uncertainty (e.g. confidence intervals)
	☒	For null hypothesis testing, the test statistic (e.g. *F*, *t*, *r*) with confidence intervals, effect sizes, degrees of freedom and *P* value noted<br>*Give P values as exact values whenever suitable.*
☒		For Bayesian analysis, information on the choice of priors and Markov chain Monte Carlo settings
☒		For hierarchical and complex designs, identification of the appropriate level for tests and full reporting of outcomes
	☒	Estimates of effect sizes (e.g. Cohen's *d*, Pearson's *r*), indicating how they were calculated

Our web collection on statistics for biologists contains articles on many of the points above.

Software and code

Policy information about availability of computer code

Data collection	NGS Data was collected by software which was offered by the manufacturer, converted into csv files using Microsoft excel 365 proplus and analyzed by publicly available python script.
Data analysis	Cell viability data analysis was performed using GraphPad Prism software version 8.2.1

For manuscripts utilizing custom algorithms or software that are central to the research but not yet described in published literature, software must be made available to editors and reviewers. We strongly encourage code deposition in a community repository (e.g. GitHub). See the Nature Research guidelines for submitting code & software for further information.

Data

Policy information about availability of data

All manuscripts must include a data availability statement. This statement should provide the following information, where applicable:

- Accession codes, unique identifiers, or web links for publicly available datasets
- A list of figures that have associated raw data
- A description of any restrictions on data availability

NGS data is available in the NCBI Sequence Read Archive (BioSample accession: SAMN17137021).
Count reads supporting all the terrace and DRACO plots, as well as data that supports the analysis presented in Figs. 2e and 8b are all available in the supplementary spreadsheets. All the other raw data-sets are available from the corresponding author upon request.

Field-specific reporting

Please select the one below that is the best fit for your research. If you are not sure, read the appropriate sections before making your selection.

☒ Life sciences ☐ Behavioural & social sciences ☐ Ecological, evolutionary & environmental sciences

For a reference copy of the document with all sections, see nature.com/documents/nr-reporting-summary-flat.pdf

Life sciences study design

All studies must disclose on these points even when the disclosure is negative.

Sample size	NGS for samples from the genome-scale screening: two independent sample sets for T0 and single sample for T1, T2, T3. Each cell viability plot is a representative of 2-5 independent experiments. T-test used for verifying the statistical significance of the experiments.
Data exclusions	No data was excluded in this study
Replication	2-5 independent experiments for cell viability assessment showing similar results. Positive experiments were usually subjected to more repeats.
Randomization	n/a
Blinding	All measurements were collected directly from the equipment and then subjected to data analysis, therefore blinding was not applicable . No subjective assessment were made.

Reporting for specific materials, systems and methods

We require information from authors about some types of materials, experimental systems and methods used in many studies. Here, indicate whether each material, system or method listed is relevant to your study. If you are not sure if a list item applies to your research, read the appropriate section before selecting a response.

Materials & experimental systems

n/a	Involved in the study	
☐	☒	Antibodies
☐	☒	Eukaryotic cell lines
☒	☐	Palaeontology and archaeology
☒	☐	Animals and other organisms
☒	☐	Human research participants
☒	☐	Clinical data
☒	☐	Dual use research of concern

Methods

n/a	Involved in the study	
☒	☐	ChIP-seq
☒	☐	Flow cytometry
☒	☐	MRI-based neuroimaging

Antibodies

Antibodies used	PRIMARY: c-Jun (60A8) Rabbit mAb #9165 CST. HSP90 (C45G5) Rabbit mAb #4877 CST. Secondary: anti-rabbit, 7074S CST.
Validation	The antibodies used in this study were validated by the manufacturer.

Eukaryotic cell lines

Policy information about cell lines

Cell line source(s)	MCF-7, T47D, BT474, ZR75-1, MDAMB231, Panc1, LnCAP
Authentication	All cell lines were originally received from ATCC
Mycoplasma contamination	All cell lines are routinely checked for mycoplasma contamination
Commonly misidentified lines (See ICLAC register)	n/a
